# The Sulfate-Rich and Extreme Saline Sediment of the Ephemeral Tirez Lagoon: A Biotope for Acetoclastic Sulfate-Reducing Bacteria and Hydrogenotrophic Methanogenic Archaea

**DOI:** 10.1155/2011/753758

**Published:** 2011-09-11

**Authors:** Lilia Montoya, Irma Lozada-Chávez, Ricardo Amils, Nuria Rodriguez, Irma Marín

**Affiliations:** ^1^Biología Molecular de Plantas, Instituto Potosino de Investigación Científica y Tecnológica, Camino a la Presa San José 2055, Lomas 4a Sección, 78216 San Luis Potosí, SLP, Mexico; ^2^Centro de Biología Molecular, CSIC Universidad Autónoma de Madrid, Cantoblanco, 28049 Madrid, Spain; ^3^Interdisciplinary Center for Bioinformatics and Department of Computer Science, University of Leipzig, 04107 Leipzig, Germany; ^4^Centro de Astrobiología, INTA, 28855 Torrejón de Ardoz, Spain

## Abstract

Our goal was to examine the composition of methanogenic archaea (MA) and sulfate-reducing (SRP) and sulfur-oxidizing (SOP) prokaryotes in the extreme athalassohaline and particularly sulfate-rich sediment of Tirez Lagoon (Spain). Thus, adenosine-5′-phosphosulfate (APS) reductase *α* (*aprA*) and methyl coenzyme M reductase *α* (*mcrA*) gene markers were amplified given that both enzymes are specific for SRP, SOP, and MA, respectively. Anaerobic populations sampled at different depths in flooded and dry seasons from the anoxic sediment were compared qualitatively via denaturing gradient gel electrophoresis (DGGE) fingerprint analysis. Phylogenetic analyses allowed the detection of SRP belonging to Desulfobacteraceae, Desulfohalobiaceae, and Peptococcaceae in *∂*-proteobacteria and Firmicutes and SOP belonging to Chromatiales/Thiotrichales clade and Ectothiorhodospiraceae in *γ*-proteobacteria as well as MA belonging to methylotrophic species in Methanosarcinaceae and one hydrogenotrophic species in Methanomicrobiaceae. We also estimated amino acid composition, GC content, and preferential codon usage for the AprA and McrA sequences from halophiles, nonhalophiles, and Tirez phylotypes. Even though our results cannot be currently conclusive regarding the halotolerant strategies carried out by Tirez phylotypes, we discuss the possibility of a plausible “salt-in” signal in SRP and SOP as well as of a speculative complementary haloadaptation between salt-in and salt-out strategies in MA.

## 1. Introduction

Molecular oxygen is found only in those biotopes that harbor organisms carrying out oxygenic photosynthesis. In oxygen-deficient systems, the nature of the redox couple and concentrations of electron acceptor/donor determine the succession of dissimilatory metabolisms due to thermodynamic conditions [1]. For a given substrate and under standard conditions, the aerobic dissimilatory metabolisms provide about one order of magnitude more energy than the anaerobic ones, for example, glucose respiration (Δ*G*°′ = −2877 kJ/mol) versus glucose fermentation (Δ*G* ^°′^ = −197 kJ/mol) [2]. Therefore, in sedimentary environments oxygen is exhausted at deeper layers and the dissimilatory metabolisms are anaerobic as a result. Anaerobic microorganisms are of interest in extreme environments because environmental parameters such as temperature and salinity regulate the rates of organic matter remineralization [3]. Extreme halophilic microorganisms require at least 15% NaCl and tolerate up to 35% NaCl. Interestingly, the low activity of water and the expense on biosynthesis only select heterotrophs and strict aerobes as extreme halophiles. However, some moderate halophilic and strict anaerobes have been described; one example is the methanogen *Methanohalobium evestigatum, *which uses methylated compounds (e.g., methylamine and methanol) to generate methane. Methylated substrates yield more energy (Δ*G*°′ = −78.7 to −191.1 kJ per mol substrate) than H_2_/CO_2_ (Δ*G*°′ = −34 kJ/mol substrate) or acetate (Δ*G*°′ = −31 kJ mol substrate) and allow that methylotrophic methanogens such as *M. evestigatum* can tolerate up to 29.2% NaCl [4]. Differences in bioenergetic yield determine an exclusion of hydrogenotrophic methanogens such as *Methanocalculus halotolerans,* which tolerates a lesser salinity: up to 12% NaCl [5]. A similar pattern has been described for sulfate-reducing prokaryotes: acetoclastic sulfate reducers (Δ*G*°′ = −47.6 kJ mol substrate), most of them belonging to *Desulfobacteraceae,* cease to tolerate high osmolarity conditions, for example, *Desulfobacter halotolerans* grows up to 13% NaCl [6]; on the other hand, *Desulfohalobiaceae* members have higher salt tolerances (up to 25% NaCl) and grow with H_2_/CO_2_ (Δ*G*°′ = −152.2 kJ mol) or lactate (Δ*G*°′ = −160 kJ/mol). 

To define whether extremes of salinity are relevant in composition and persistence of anaerobic ecotypes, the ephemeral systems and spatial gradients constitute appropriate sites of study. Even though there are some studies about microbial communities present along salt gradients, those approaches have been performed on thalassic microbial mats [7]; therefore, they are depleted in sulfate at deep layers, but most of them are also formed only on intertidal zones. Sulfate is the second most abundant electron acceptor on Earth and consequently the dominant electron acceptor for anaerobic metabolism in marine sediments [8]. One interesting ephemeral and sulfate-rich system is Tirez lagoon, or *sabkha,* because it is subjected to flooding/desiccation regimes, located in “La Mancha,” an arid region in Spain. Tirez lagoon is athalassic since the ionic composition is far from seawater and it is characterized by a low Cl : SO_4_ ratio (about 1.18 in flooded season and 0.35 in the dry season), whilst in the Dead Sea this ratio is above 10^3^ [9]. This system is maintained at a neutral pH due to a high Mg^2+^ and Ca^2+^ concentration in combination with a low CO_3_^2−^ content at the saltern and sediment environments. The traces of CO_3_^2−^ are removed as dolomite (CaMg(CO_3_)_2_) preventing alkalinization [10]. Given this scenario, the primary objective of this study was to characterize the composition of the anaerobic populations in the ephemeral and sulfate rich Tirez Lagoon. 

The identification of environmental sulfate reducing prokaryotes (SRP) and sulfur oxidizing prokaryotes (SOP) can be performed by enrichment culturing and molecular ecology fingerprinting; however, a characterization of methanogenic archaea (MA) through isolation techniques is problematic given their slow growth rates [11]. The use of molecular ecology fingerprinting techniques such as denaturing gradient gel electrophoresis (DGGE) from PCR-amplified genes is informative to assess the temporal and spatial qualitative diversity in natural samples, and it also requires fewer sequencing resources in comparison to clone libraries and/or metagenomic analysis [12]. Instead of the 16S rRNA gene, the use of DGGE from PCR-amplified functional gene markers is profitable to elucidate the composition of the anaerobic pathways of sulfate respiration (SR), sulfur oxidation (SO), and methanogenesis (MT). The 16S rRNA gene-based analysis cannot provide an unambiguous link between gene sequences and its physiological or metabolic role [13]. 

Whereas the SRP and SOP organisms are phylogenetically and physiologically disperse along the *Bacteria *and *Archaea *domains [14], MA organisms are monophyletic restricted to* Archaea *[15]. In the dissimilatory pathways of sulfate reduction and sulfur oxidation, dissimilatory sulfite reductase (*Dsr*) and adenosine-5′-phosphosulfate (APS) reductase (*Apr*) are considered as key enzymes [14]. In the sulfate-reducing pathway, sulfate has to be activated to APS by ATP-sulfurylase (EC: 2.7.7.4) at the expense of ATP; *Apr* (EC: 1.8.99.2) converts the APS to sulfite and AMP; hereafter, sulfite is reduced to sulfide by *Dsr* (EC: 1.8.7.1). For the sulfur-oxidizing pathway, the reverse direction is operated by homologous and conserved enzymes [16]. The alpha subunits of *Apr* and *Dsr* enzymes are found in all known SRP and most of SOP [17]. Regarding the methanogenesis pathway, the methyl coenzyme-M reductase (*Mcr*) (EC: 2.8.4.1) catalyses the reduction of a methyl group bound to coenzyme-M, with the concomitant release of methane [15]. *McrA* is unique and ubiquitous in all known MA [18]. *McrA* gene fragment provides more information than the 16S rRNA gene; even if the saturation rates are similar between the *McrA* gene fragment and the complete 16S rRNA gene, the number of differences per site in the *McrA* fragment is 2-3 times higher than that in the full-length *16S rrs* [19]. Therefore, assignment of genera with *McrA* sequences offers more conclusive resolution than assignment with 16S rRNA gene sequences. The mutation rates and selective pressures of the *AprA* and *McrA* metabolic gene markers and of the structural 16S rRNA gene are different; however, phylogenetic studies done with partial sequences of *AprA* and *McrA *belonging to the SRP, SOP, and MA lineages have established an agreement with the phylogenetic relationships based on 16S rRNA gene sequences [13, 18]. Therefore, these functional gene markers can provide an estimate of the SR, SO, and MT microbial diversity harbored in sediments of Tirez Lagoon. Indeed, databases have been enriched in sequences of model strains for these two enzymes; as a consequence, the *aprA* and *mcrA* gene markers also provide us information to identify SRP, SOP, and MA selectively in complex microbial communities, for example, [20].

The second aim of this study was to investigate whether the composition and distribution of the encoded amino acids in *aprA* and *mcrA* genes are indicative of haloadaptation to the hypersaline sediment. Diverse lines of evidence report that halophilic microorganisms can bias their amino acid composition to deal with the multimolar salinities of their environment [21, 22]. This adaptative and energetically efficient strategy is characteristic in “salt-in” halophiles, where turgidity is maintained by the intracellular accumulation of K^+^ that is usually equilibrated with the presence of extracellular Cl^−^ [23]. Therefore, an increase of the acidic nature of cytoplasmic proteins, which is offset by an overall decrease in basic amino acids, is needed to maintain an appropriate folding and functionality under osmotic stress [22, 24]. In cytoplasmic proteins, it has been also pointed out a slight decrease in hydrophobicity as another amino acid haloadaptation [25, 26]. In contrast, “salt-out” halophiles build up concentrations of osmolytes (also named osmoprotectants or compatible solutes) to increase the intracellular osmolarity; thereby maintaining the protein native states in spite of a highest energetic cost to manufacture the organic molecules [27]. Accordingly, only proteins in “salt-out” organisms exposed directly to the hypersaline medium exhibit an excess of acidic amino acids [28]. All eukaryotes, most halophilic bacteria, and the halophilic methanogenic archaea (such as *Methanohalobium evestigatum*) have evolved the “salt-out” strategy [21]. The widely disparate taxonomic position of “salt-in” prokaryotes (Halobacteriales in Archaea, *Salinibacter ruber,* and Halanaerobiales in Bacteria) suggests a convergent evolution of this osmoadaptation strategy [27].

Several studies have also reported that a high genomic CG content (often upwards of 60%) and a GC bias at the codon usage level are common adaptations to hypersaline environments, presumably to avoid UV-induced thymidine dimer formation and accumulation of mutations [21, 23]. For example, the high GC composition (65.9%) of *Halobacterium sp.* NRC1 could reduce the chance of such lesions and its third position GC bias correlates with an overrepresentation of acidic residues (i.e., Asp and Glu) [29]. The unique exception to this general trend has been pointed out so far in another extreme halophile *Haloquadratum walsbyi* shows a remarkably low genomic GC content (47.9%) and a weak GC-bias at the codon usage level [30]. Given that other specific features of nucleotide selection may also be involved in the GC content of organisms, the GC-bias measurements are complementary to the amino acid composition but not decisive in order to infer the “salt-in” strategy [25]. 

Therefore, the findings of this study try to contribute to the knowledge of diversity and haloadaptation of the SRP and MT thriving at rich sulfate sediment. Additionally, Tirez system is analog to the ocean of Europa, satellite of Jupiter, due to its sulfate abundance and neutral pH [31], and sulfates have been detected on Mars indeed [32]. Thus, this knowledge will provide insight regarding the possible biological limits for life in other analogous places.

## 2. Materials and Methods

### 2.1. Study Site and Sampling Procedure

Sediment samples were collected from Tirez lagoon, which has an area of <1 km^2^ and it was originated after endorheic inflow under semiarid conditions. The lagoon is located in the southern subplateau of the Iberian region of La Mancha (39° 32′ 42′′ N y 03° 21′ O). The salt content fluctuates from 6% (w/v) during winter to 35% (w/v) during spring. In summer, the system becomes an evaporite. Temperature oscillation is about 40°C and −7°C, the mean annual thermal oscillation is 55°C, and the annual mean rainfall is averaged at 400 mm. Water drains through material from the Triassic period; dolomites and Ca-sulfate marls are from the Tertiary period [31]. 

Samples were collected in February and July 2005 and correspond to the winter and summer seasons, respectively. The winter and summer samplings were done by triplicate in three points at the lagoon; all of them were located in the salt pan or lagoon basin because it is the region covered by salts in summer. The sample cores were obtained from sites separated from each other by several meters. In order to analyze seasonal changes in the lagoon, the summer samples were obtained from the holes-signals leaved by the winter (flooded) sampling. Sediment cores were obtained with a Ring Kit core-sampler for soft soil to a depth of 40 cm. The sampled cores were cooled at −20°C with jelly bags and kept until further processing.

### 2.2. Physicochemical Parameters

The sediment cores were sampled in winter and used to perform physicochemical analyses. Eh and pH of the cores were measured with a probe connected to a potentiometer Orion Model 290A + Thermo Orion (Thermo Fisher Scientific). Also, dissolved oxygen and temperature were measured with a Sylant Simplair F-15 oxymeter (Syland Scientific GmbH). In order to determine the interstitial sulfide concentration, the sample cores from surface to 20 cm in depth were sonicated for 5 min (Labsonic B. Braun sonicator). After centrifugation (14,000 rpm 10 min Sorvall RC-5), supernatants were mixed with Zn acetate (2%) and sulfide concentration was determined using the methylene blue method [33]. The core samples used to analyze ion content (Cl^−^, SO_4_^2−^, and NH_4_^+^) and carbon : nitrogen (C : N) ratios were sampled from the surface to 20 cm in depth; samples were dehydrated at 110°C for 12 hrs for ionic chromatography and elemental analysis. Ionic chromatography analysis was completed with 100 mg of pulverized samples diluted in 25 mL of filtered milliQ water, whilst the elemental analysis was performed with dried and pulverized sediment. The sediments were assayed by chromatographic methods with an IC Dionex DX-600 chromatograph and by spectrophotometric methods with a LECO CHNS-932 elemental analyzer at the *Servicio Interdepartamental de Investigación* (UAM).

### 2.3. Enrichment of SRP

SRP organisms from winter samples were grown in a cysteine-reduced (4.12 mM) medium for sulfate reducers, modified from Raskin et al. [34], and contained glutamic acid (5.2 mM), glycine (0.2 mM), methanol (14 mM), methylamine (27 mM), peptone (250 mg/L), and yeast extract (250 mg/L). The salt content in sulfate-reducing media was 3.5% the Tirez saltern. The inoculation was done with sedimentary slurry from samples collected in February 2005 (winter season). Cell culture growth was monitored through the count of the cell density with 4′,6-diamino-2-phenylindole (DAPI), Molecular Probes (Invitrogen) [35] in a Zeiss Axiovert 200M fluorescent microscope. Sulfide increase was also followed [33] for 12 months of incubation at 30°C. Nonaxenic cultures were subjected to DNA extraction.

### 2.4. DNA Extraction

Core samples were cut with sterile surgical blades according to depth regions. The three cores with a weight of ~210 g were mixed with three volumes of PBS 1x at 4°C to reduce microheterogeneities and to wash salts. This mixture was sonicated for 3 min (Labsonic B. Braun sonicator). Integrity of bacterial cells after the treatment was confirmed by 4′,6-diamino-2-phenylindole (DAPI) 1 *μ*g/mL. Total genomic DNA was extracted from supernatants of washed and centrifuged sediments (500 rpm for 1 min Hettich Mikro 22 R centrifuge to precipitate rocks). In order to collect cells from nonaxenic cultures, 100 mL of the samples were filtered onto 0.22 *μ*m of polycarbonate filters (Millipore). Sediment and soil samples are characterized by the presence of inhibitors such as humic acids and exopolimeric substances, thus we used a specialized DNA extraction kit (*FAST DNA SPIN kit for soil)* (QBiogene, Irving, Calif, USA) which has proved to retrieve a reliable DNA extraction to obtain a broad and intense band patterns, in comparison with variants via phenol DNA extraction [36], and it has been used for analyses of microbial diversity by DGGE in sediments, for example, [20]. Total genomic DNA was purified according to Genomic DNA purification JetQuick kit (Genomed) instructions. 

### 2.5. PCR Amplification

Amplifications were carried out in a Thermal Cycler 2720 (Applied Biosystems). PCR reactions were performed in a mixture of 50 *μ*L containing: 2 *μ*L of template DNA, 1 mM dNTP'S, 0.5 *μ*M of each primer, 3 mM MgCl_2_, 1x enzyme buffer, and 0.03 U/*μ*L AmpliTaq DNA Polymerase (Roche, Molecular Systems). The *aprA* gene fragment of ~0.4 kb was amplified with the APSfw (TGGCAGATMATGATYMACGGG with a GC clamp) and APSrv (GGGCCGTAACCGTCCTTGAA) primer pairs. The following conditions were implemented: a first denaturing step at 94°C for 3 min, the completion of 35 cycles of 30 s at 94°C, an annealing at 60°C for 55 s and at 72°C for 1 min, and a final extension of 72°C for 7 min [37]. In the *mcrA* gene fragment amplification, at first a 0.76 kb fragment was amplified with the primers ME1 (GCMATGCARATHGGWATGTC) and ME2 (TCATKGCRTAGTTDGGRTAGT). The ME-PCR reaction was carried out with an initial denaturing step at 94°C (5 min), followed by 25 cycles of 1 min at 94°C, an annealing at 57°C for 1 min and at 72°C for 2 min, and a final extension of 72°C during 10 min [38]. ME-PCR product was used as template to amplify an internal 0.47 kb fragment (Figure S4 see in supplementary Material available at doi: 10.1155/2011/753758). Nested PCR was performed with the primers MLf (GGTGGTGTMGGATTCACACARTAYGCWACAGC) and MLr (TTCATTGCRTAGTTWGGRTAGTT) with a GC clamp applying the following conditions: a denaturing step of 5 min at 94°C, 5 cycles at 95°C for 40 s, 55°C for 1 min, 72°C for 90 s (a ramp of 0.1°C/s was included between the annealing to the extension steps), followed by 30 cycles of 95°C for 40 s, 55°C for 1 min, 72°C for 90 s, and an extension of 72°C for 7 min [18]. The GC clamp was equivalent to 40 bp of GC at the 5′ end in order to prevent a complete melting of the DNA fragments. Correct length PCR-DGGE products were visualized on 0.5 *μ*g/mL ethidium-bromide-stained gels at 2% agarose.

### 2.6. Denaturing Gradient Gel Electrophoresis (DGGE)

In order to generate a DGGE pattern, an average of 50–70 *μ*g of DNA from PCR-DGGE products were resolved using a D-Code Universal Mutation Detection System (BioRad Laboratories) in polyacrylamide gels with a horizontal denaturant gradient. All DGGE patterns were achieved under standardized denaturant and electrophoretic conditions: constant temperature of 60°C polyacrylamide composition (acrylamide-N,N′-methylene bisacrylamide, 37 : 1) containing 0–100% of denaturants (7 M urea and 40% formamide deionized with mixed-bed resin), a running time of 4.5 hrs and a constant voltage of 200 V [39]. High resolving band patterns from environmental and culture samples were obtained as follows (denaturant composition is given in percentage): for *aprA *gene fragments, 50–80% from nonaxenic cultures, and 40–70% from environmental samples. In *mcrA* gene fragments from environmental samples, the performed gradients were done by duplicate at 40–70% and 40–60% to increase the resolution of distance among bands. The gels were incubated in ethidium bromide for 20 min and rinsed in distilled water for 30 min. All single bands were excised from the gel with scalpels and eluted in 10 *μ*L of milliQ water to avoid desiccation; hereafter, they were stored overnight at 4°C. DNA was extracted from polyacrylamide by electrophoresis in 2% agarose gels (≤40 mA). The agarose bands were filtered through glass fiber columns at 14,000 rpm for 2 min (Hettich Mikro 22 R centrifuge). 5–10 *μ*L of the precipitate obtained were used as DNA template for the band reamplification of *mcrA* and *aprA* genes under the same PCR conditions however, a minor fraction of the bands were reamplified. The *mcrA* and *aprA* gene PCR products were sequenced using primer pairs APSfw/APSrv and MLf/Mlr in an ABI 377 sequencer (Applied Biosystems). Nucleotide sequences were cleaned and assembled using DNA Baser software (Heracle Software, Germany, http://www.DnaBaser.com/). 

### 2.7. DGGE Band Pattern Analysis

According to the review of Fromin et al. [40], DGGE reproducibility mainly relies on the DNA extraction and/or PCR amplification steps; therefore, the fingerprint analysis (DGGE) was processed once. The reproducibility of DGGE patterns has been tested previously by experimenting differences along the procedure, from sampling to PCR amplification conditions; despite these modifications, the comparison of DGGE patterns was consistent showing changes in band intensities only [41]. Thereby, the pattern of *aprA* DGGE gel from environmental samples was used to define a dendrogram. Then, the bands were qualitatively scored as present/absent and no semiquantitative analyses were performed for band intensity; the band clustering was performed with the maximum likelihood (ML) restriction analysis (RESTML) included in the PHYLIP v.3.67 package [42].

**GenBank Accession Numbers**

The nucleotide sequence data reported here are available under the GenBank accession numbers: EU722715–EU722732, HM466937–HM466940, HM466943–HM466946 (*aprA* phylotypes), and EU091355–EU091364, HM466948 (*mcrA* phylotypes).

### 2.8. Phylogenetic Analysis

The translations of the *aprA* and *mcrA* sequences into amino acids were defined using the TRANSLATE tool with a standard code (http://expasy.org/tools/dna.html/). The best frames for all the *aprA *and *mcrA* fragments were firstly selected by the unstopped amino acid (aa) sequences and, secondly, by matching their best hits with those compiled in the nonredundant database of the GenBank, which were detected through the BLASTP program (*e*-value ≤ 10^−3^) [43]. The final inferred aa sequences were compared against the Swiss Prot and GenBank databases in order to obtain their homologous counterparts using the WU-BLAST program [44] with a significant BLASTP *e*-value ≤ 10^−3^. From a first approach, we also included *aprA* and *mcrA* sequences from reported environmental samples as seed sequences. A complete list of the sequences included in this study to reconstruct a phylogeny for the *AprA* and *McrA* enzymes can be seen in Supplementary Material (Tables S1 and S2, resp.).

Different filters were used, from the thousands of collected sequences, in order to choose the final candidates involved in the reconstruction of a phylogenetic hypothesis. In this sense, we firstly applied a redundant analysis at 90% identity using the CD-HIT program [45]. From the obtained sequences, a second analysis at 100% identity was done with the CD-HIT program, which excludes redundant phylotypes (subspecies and variants) of the same species, warranting the diversity of sequences only by including strictly different species from the same or different genera. The *AprA* phylotypes aps cw 1 (EU722715) and aps cw 16 (EU722724) showed 100% of identity, as well as the phylotypes aps cw 3 (EU722716) and aps cw 10 (EU722720). Only one sequence was taken as a representative of the identity cluster to reconstruct the phylogeny. In order to support a robust identification in the final phylogeny, we included species from the same genera for those cases in which the homologous counterparts are closely related to the phylotypes obtained in this work. When it was necessary, individual phylogenetic trees for the Tirez phylotypes were done previously in order to improve their identification and to select the counterpart sequences for the final phylogeny. The aa sequences obtained from the previous approaches were then aligned using the CLUSTALX program with default parameters [46]. In order to identify an evolutionary signal from the sequence fragments obtained in this work and their homologous counterparts, we applied a manual and also an automatic approach to edit the alignment.

First, we manually edited the alignment through the use of the BIOEDIT program v.7.0.9 [47] in order to include only the functional domains of the *α* subunits of *mcr *and *apr* enzymes. The functional description of these domains is fully detailed in Section 4 and in the Supplementary material. The N-terminal domain of the *α*-subunit of the *AprA* (AprA_alpha_N) harbors the FAD cofactor-binding domain (aa positions: 2–261 and 394–487) and the capping domains (aa positions: 262–393). These functional domains have been characterized from* Archaeoglobus fulgidus* in the reduced state (FAD_red_-APS, PDB ID: 1JNR) [16] and in the oxidized state (FAD_ox_-APS, PDB ID: 2FJA) [48] as well as in *Desulfovibrio gigas *(PDB ID: 3GYX) [49]. Thus, a total of 100 aa sequences were included in the *AprA* alignment, where 23 phylotypes are derived from this work. The *AprA* alignment includes two of the nine (the absent sites are Asn-N^*α*74^, Tyr-Y^*α*95^, Glu-E^*α*141^, Val-V^*α*273^, Gly-G^*α*274^, Leu-L^*α*278^, and Arg-R^*α*317^) functional active sites of the AprA_alpha_N domain: Arg-R^*α*265^ and Trp-W^*α*234^, previously reported [48]. See the *AprA* alignment and catalytic sites in Supplementary Material (Figure S2). On the other hand, the C-terminal domain of the *α*-subunit of the *McrA *enzyme (Mcr_alpha_C) harbors an all-alpha multihelical bundle domain (PFAM domain: PF02249). This functional domain has been characterized in *Methanosarcina barkeri* (PDB ID: 1E6Y, C-terminal domain: 328–460) [50], *Methanothermobacter thermoautotrophicus* (PDB ID: 1MRO, C-terminal domain: 315–440) [51], and *Methanopyrus kandleri*(PDB ID: 1E6V, C-terminal domain: 319–444) [52]. A total of 80 aa sequences, 11 of them derived from this work, were included in the *McrA* alignment. The *McrA* alignment includes five of the seven functional active sites of the Mcr_alpha_C domain (absent sites: Asn-N^*α*481^ and Val-V^*α*482^; present sites: Phe-F^*α*330^, Tyr-Y^*α*333^, Phe-F^*α*443^, Tyr-Y^*α*444^, and Gly-G^*α*445^) [51]. See the *McrA* alignment and catalytic sites in the supplementary material (Figure S3). 

Finally, we readjusted a final alignment defining the informative sites and conserving the functional active sites of the enzymes previously described, through the use of the software GBLOCKS v.0.91 [53]. Therefore, the final alignments were performed on a region of 137 aas for *AprA* and 139 aas for *MrcA*, from which 122 and 132 positions were involved in the phylogenetic analysis, respectively. In order to reconstruct a phylogenetic tree, a character-based approach for the Apr_alpha_N and Mcr_alpha_C phylogenetic reconstructions was developed using the PROTPARS program in order to construct a maximum parsimony (MP) tree of sequences. *Pyrobaculum aerophilum* and* Archaeoglobus fulgidus *were used as outgroups in the Apr_alpha_N phylogeny; whereas *Methanopyrus kandleri* was used as outgroup in the Mcr_alpha_C phylogeny. A distance approach for the Apr_alpha_N and Mcr_alpha_C phylogenetic reconstructions was also developed using the SEQBOOT program to generate 1000 bootstrapped datasets from the sequences, whereby the pseudoreplicates were used in the PROTDIST program in order to generate a distance matrix through the Jones-Taylor-Thornton (JTT) model of evolution [54]. The evolutionary distances are in the units of the number of amino acid substitutions per site. The rate variation among sites was modeled with a gamma distribution estimated previously shape parameter = 0.9 for *AprA* and = 0.8 for *McrA*. Then, the distance matrix was used in the NEIGHBOR program to construct a neighbor-joining (NJ) tree [55]. The bootstrap consensus tree inferred from 100 replicates is taken to represent the evolutionary history of the taxa analyzed. Branches corresponding to partitions reproduced in less than 60% bootstrap replicates were collapsed with the CONSENSUS program (default parameters). The percentage of replicate trees (>50%) in which the associated taxa clustered together in the bootstrap test (100 replicates) is shown next to the branches. All these programs belong to the PHYLIP package v.3.68 [42]. No major branching differences were detected between the MP and NJ topologies obtained for both enzymes. The trees were visualized and reannotated using the MEGA and Microsoft Photoshop programs [56].

### 2.9. Amino Acid Composition Analysis

We calculated the amino acid composition of all AprA and McrA sequences used previously to construct the phylogenies. Using the Perl scripting language, a program was written to read each amino acid sequence (FASTA format) and calculate the frequency for each amino acid. We also calculated two halophilia indicators from the amino acid composition of every sequence: the PAB factor estimates the surplus of polar and acidic amino acid compared to polar and basic ones (i.e., PAB = [Asx + Glx] − [Arg + Lys]) [57], and the AB ratio of the acidic amino acids Glu and Asp to the basic amino acids Lys, His, and Arg (i.e., AB = [Asp + Glu] : [His + Arg + Lys]) [21]. We divided the amino acid profiles from each marker into two different data sets in order to calculate an average and standard deviation of the samples. The first data set is based on salinity adaptation by dividing sequences in Tirez, halophilic, and nonhalophilic species. The second data set is based on the species forming the major taxonomic groups in which the Tirez phylotypes are phylogenetically allocated. For AprA Desulfovibrionales, Desulfobacterales, Peptococcales, and Chromatiales; whilst for McrA, Methanomicrobiales and Methanosarcinales. Therefore, A spreadsheet was created using Microsoft Excel software for data tabulation and graph construction. See Supplementary Material. 

### 2.10. GC Content and Codon Bias Analyses

We performed the corresponding nucleotide alignments for all AprA and McrA sequences used previously to construct the phylogenies. In order to reduce the bias of the GC measurements by the use of sequences with different length and highly divergent regions (i.e., long indels), we manually edited and readjusted the final alignment. Accordingly, highly and long divergent regions (insertions and deletions) were eliminated from the alignment. The final alignments only include the strict codon positions encoding for the functional domains of AprA (375 nucleotide positions, 125 codons) and McrA (399 nucleotide positions, 133 codons) described on the phylogenetic analysis section. Using the Perl scripting language, a program was written to read each nucleotide sequence (FASTA format) and calculate the total nucleotide percentages as well as at the three individual codon positions for each sequence. We divided the GC profiles from each gene marker into the same data sets used to analyze amino acid composition (i.e., salinity adaptation and taxonomic clades) in order to calculate an average and standard deviation of the samples. A correction for the amino acid usage was applied by the calculation of the relative synonymous codon usage (RSCU) values from the nucleotide datasets based on salinity adaptation: Tirez, halophilic, and nonhalophilic species. The RSCU for a particular codon (i) is given by: RSCU_*i*_ = *X*_*i*_/∑∑  *X*_*i*_/*n*, where *X*_*i*_ is the number of times the codon has been used for a given amino acid and *n* is the number of synonymous codons for that amino acid. RSCU values are the number of times a particular codon is observed, relative to the number of times that the codon would be observed in the absence of any codon usage bias [58]. In the absence of any codon usage bias, the RSCU value would be 1.00. A codon that is used less frequently than expected will have a value of less than 1.00 and *vice versa* for a codon that is used more frequently than expected. Finally, a spreadsheet was created using Microsoft Excel software for data tabulation and graph construction. See supplementary material. 

## 3. Results

### 3.1. Physicochemical Characterization

**Figure 1:**
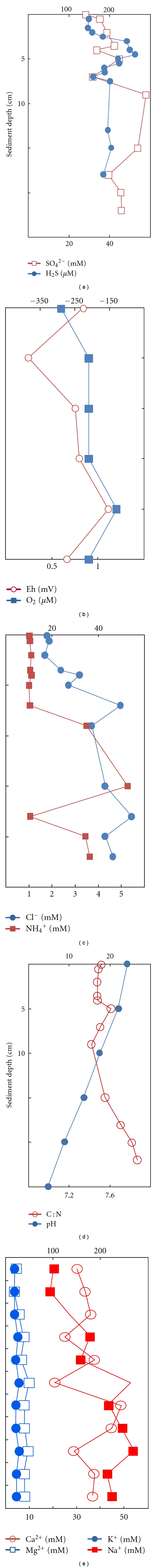
Profiles plotting depth against physicochemical parameters measured in Tirez sediments from winter cores. (a) Sulfide and sulfate; (b) Redox potential (Eh) and oxygen; (c) chloride and ammonium; (d) pH and C : N ratio; (e) magnesium, calcium, potassium, and sodium.

**Figure 2:**
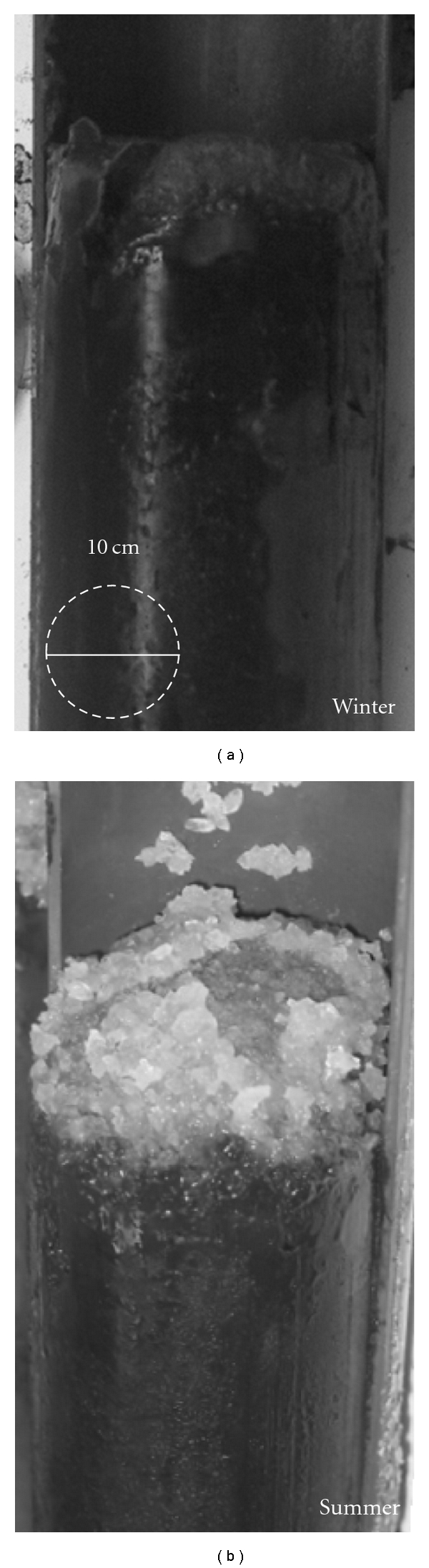
Cores of sediments of Tirez ephemeral lagoon for analysis collected from winter and summer showing the dark zone in the upper region probably due to metal sulfide precipitation. The evaporite is founded in summer sample.

Sediment cores from Tirez Lagoon sampled at different depths were subjected to physicochemical analysis. Sulfide showed higher concentrations at the zone of 0–10 cm depth (Figure 1(a)). The occurrence and distribution of sulfide along the depth profile can reflect a biogenic origin by the presence of sulfate-reducing bacteria (SRB) in the hypersaline sediment. The concentration of H_2_S coincided with the presence of a black deposit of iron sulfide mainly in winter (Figure 2). Sulfate levels increased with depth, its concentration ranging at 0.2 M, and the highest values were detected at 10–15 cm in depth (≤300 mM), just below the highest concentration zone of sulfide (Figure 1(a)). The complete sediment profile was anoxic and in accordance with a negative redox potential (Figure 1(b)). The redox potential and oxygen levels slightly increased in the deepest zones (15–20 cm in depth). The redox conditions of most part of the sediment core were in the range of −300 and −200 mV, low enough to allow SR and MT activities [59]. The lowest Eh values were reached at 0–10 cm in depth and coincided with the increase in sulfide concentration (Figure 1(b)). Ammonium concentration fluctuated between 1 and 6 *μ*M. Likewise, the highest NH_4_^+^ concentration (4–6 *μ*M) was observed at 10–15 cm in depth (Figure 1(c)). The Cl : SO_4_ proportion fluctuated between 0.1 and 0.3, these ratios are lower than the values reported in the saltern [60] and they reflect the athalassic nature of the system. Sulfate concentration in Tirez Lagoon was lower than in the also athalassic Chaka Lake sediment (10^−1^ mM). Even though chloride was undetermined in Chaka Lake sediment, its Cl : SO_4_ proportion is two times higher than the highest value registered at Tirez Lagoon [61].  Figure 1(d) shows the pH course on sediment depth; it is possible to observe the characteristic neutral pH of the system as well as a slight acidification, probably a consequence of biological volatile fatty acids (VFA) formation and sulfate reduction processes. The C : N ratio determined in the samples showed values characteristic of low photoautotrophic activity at the surface [62] starting at >6 at 0–5 cm depth (Figure 1(d)). Therefore, preferential nitrogen mineralizers should be found at the surface preceding carbon mineralizers at deeper zones.  Figure 1(e) describes that divalent cations dominate over monovalents. Finally, the ratio (Na^+^ + K^+^)/(Mg^2+^+ Ca^2+^) in Tirez is between 1.8 and 0.09, whilst in Salt Lake is >9.0 [9].

### 3.2. DGGE Patterns from aprA and mcrA Gene Fragments

**Figure 3:**
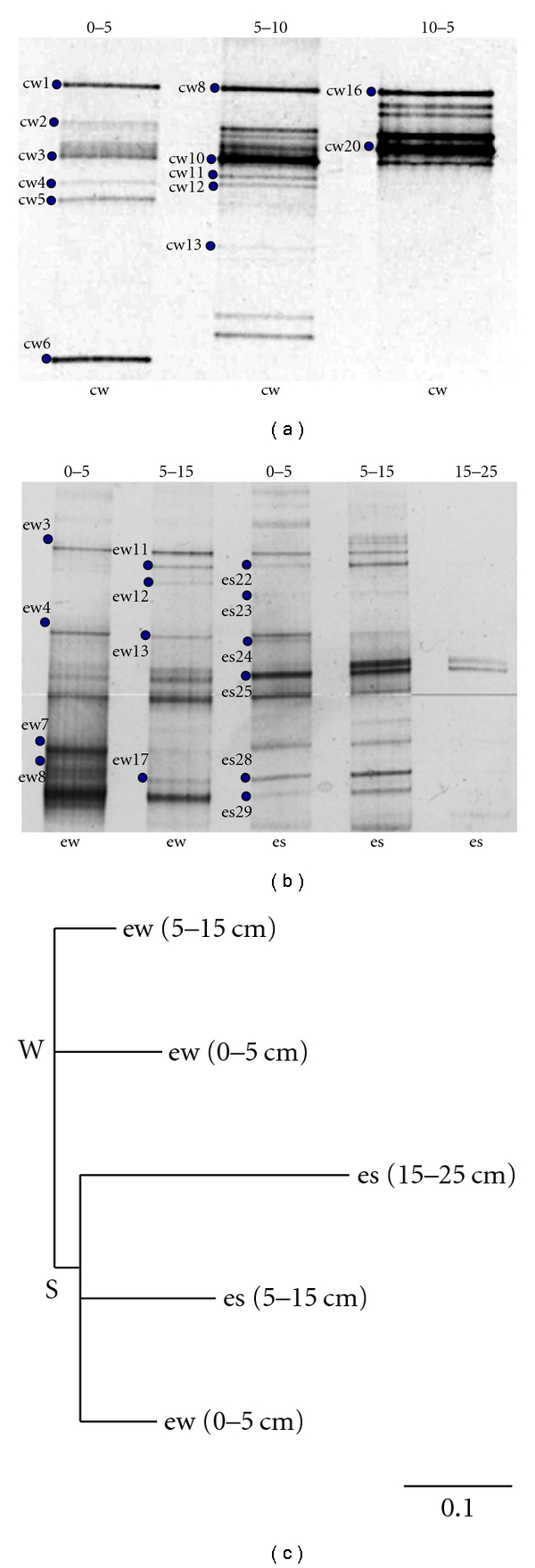
DGGE pattern of PCR-amplified *aprA *gene fragments from nonaxenic SRP cultures (a) and sediment samples (b). (a) series of DGGE patterns obtained from nonaxenic cultures inoculated from winter sediment (cw); (b) Series of DGGE patterns obtained from environmental samples: winter (ew) and summer (es) obtained from different depths (cm). The *aprA* gene fragment from 15–25 cm depth (winter) was not amplified. (c) Maximum Likelihood cluster analysis of the B-pattern DGGE profile the scale bar represents expected numbers of base substitutions.

**Figure 4:**
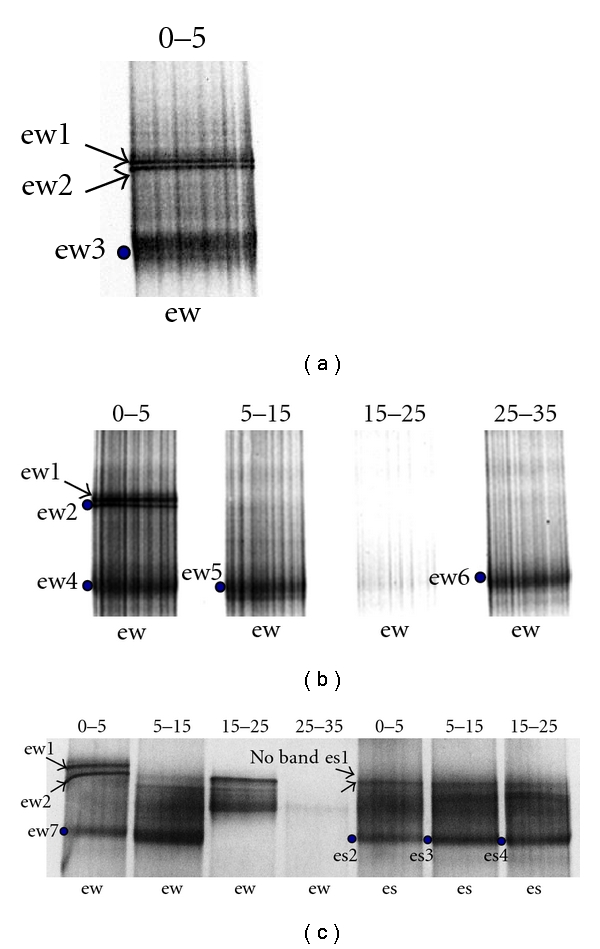
DGGE pattern of PCR-amplified *McrA* gene fragments from environmental sediment samples. (a) winter pattern (0–5 cm depth), (b) winter patterns obtained from different depths, and (c) Winter (ew) and summer (es) patterns from different depths (cm). Bands across several lanes were identified as being in the same genera, and the arrow for ew1 shows its absence in the 0–5 cm depth summer sample (es). Band mcr-es5 is not shown in the figure.

We applied a denaturing gradient gel electrophoresis (DGGE) fingerprinting analysis through the use of two functional genes: adenosine-5′-phosphosulfate reductase (*Apr*) and the methyl coenzyme-M reductase (*Mcr*), in order to identify ecotypes from the sediments samples and nonaxenic cultures of Tirez Lagoon. *AprA *DGGE profiles are presented in Figure 3 and *McrA* DGGE profile in Figure 4. Thus, we obtained sequences of diverse phylotypes from DGGE profiles representing the bulk content of three sampling points of the lagoon salt pan. The bands were prefixed as aps and mcr (from the gene marker) and subfixed as cw (from nonaxenic cultures obtained in winter) and ew and es (environmental sediment sampled in winter and summer, resp.). 

The *aprA* DGGE pattern from sediment profile (Figure 3(b)) revealed the presence of a more complex banding pattern in comparison with the profile from nonaxenic cultures (Figure 3(a)). At 15–25 cm depth, low yield or no PCR product was obtained (Figure 3(b) lane 5). Given that a considerable number of environmental bands from the *aprA *DGGE profile could not be sequenced or specifically identified, probably due to the presence of residual PCR inhibitors such as humic acids coextracted with genomic DNA [63] that were not purified by the *JetQuick kit* and that comigrate with DNA in the polyacrylamide gel [64] changes in population distribution were estimated through the use of P-analysis with Maximum Likelihood (ML) in Phylip software in order to identify a significant clustering. Bands were taken as species, and patterns were constructed by presence and absence. The clustering pattern is shown in Figure 3(c), and it was more in accordance with a disturbance due to seasonality instead of sediment depth. Additionally, *P* value showed no significant differences between nodes W and S being *P* ≤ 0.05 as significant to reject the hypothesis that two population sets were derived from the same communities. 

From previous studies carried out in thalassic communities, where salt gradient is between 8 and 20% (within the range of Tirez), it has been reported that the rate of methanogenesis is below 0.1% of the total sulfate-reduction productivity [65]. Therefore, a lower abundance of MA was expected in Tirez sedimentary community it is inferred from the lower Cl : SO_4_ ratio. In addition, the population size threshold for DGGE detection is ≤1% [39]. Thereby, we had to perform a nested PCR from the *mcrA* gene in order to improve the detection of the MA community in the sediment samples from Tirez. We firstly obtained a 0.76 kb *mcrA* fragment through the ME primer pair. Because such a length is inadequate to obtain a discernible DGGE pattern [66] and due to low yield in ME amplicons, a small 0.47 kb *mcrA* fragment nested in the ME region was amplified through the ML primer pair (supplementary material, Figure S4). In agreement with Juottonen and collaborators [67], no differences in the diversity of MA organisms were expected from the use of ME and ML PCR products. Different DGGE gradients for the ML-PCR products were tested in order to obtain the best pattern resolution. We detected two distinctive but adjacent bands in all DGGE winter profiles (e.g., mcr-ew1 and mcr-ew2) obtained through several gradients (Figures 4(a) and 4(c), 40–70% and 40–60% gradients, resp.). A pattern of bands in pairs is a result of the low DGGE resolution, where two DNA fragments differ in one or few bases due to the use of ambiguous primers [66]. Though ME-ML primers are ambiguous (see Section 2), the phylotypes were placed in different orders in methanogenic archaea. Thereby, nucleotide sequences have similar electrophoretic mobilities but they represent different sequences and, thus, a double band was ruled out. We also obtained a DGGE pattern from sediment sampled in flooded and dry seasons at different depths (Figure 4(c)). It is interesting to note that the mcr-ew1 band (marked in Figures 4(a) and 4(c)) appeared uniquely associated with flooded season at 0–5 cm in depth (Figure 4(c)). 

### 3.3. Phylogenetic Diversity of Sulfate-Reducing, Sulfate-Oxidizing and Methanogenic Organisms

Phylogenetic reconstructions were done for the inferred amino acid (aa) sequences of *aprA* and *mcrA* gene markers and their homologous counterparts. We decided to analyze aa instead of nucleotides because the latter reduces the inherent variation seen in protein sequences, except for the third codon base. We defined two regions of unambiguously aligned aa, the first one located in the N-terminal domain (137 aas) for the *α* subunit of *AprA* (AprA_alpha_N), and the second one located in the C-terminal domain (139 aas) of the *α* subunit of *McrA *(MrcA_alpha_C), both of them containing some of the catalytic sites involved in their metabolic role (supplementary material, Figures S2 and S3, resp.). It is important to note that not only the phylogenetic topologies obtained for the AprA_alpha_N and McrA_alpha_C sequences are robust, as can be seen by the significant bootstrap values in the main clustering branches, but also the internal groups are supported by the expected clustering of the *McrA* and *AprA* crystals previously characterized for (a) the *McrA* in *Methanosarcina barkeri *belonging to Methanosarcinales [50], *Methanothermobacter thermoautotrophicus* from Methanobacteriales [51] and *Methanopyrus kandleri* in Methanopyrales [52]; (b) the *AprA* from *Archaeoglobus fulgidus* in Euryarchaeota [16], and *Desulfovibrio gigas* in Deltaproteobacteria [49].

**Figure 5:**
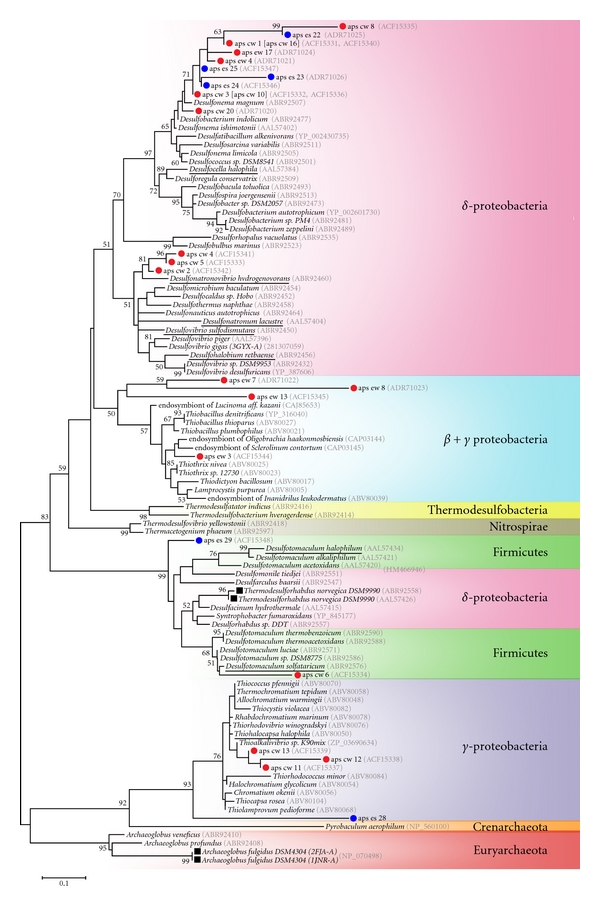
The phylogenetic tree is showing the relationship of *AprA* sequences from environmental samples and nonaxenic cultures from Tirez sediments and *aprA* sequences of characterized SRP and SOP (supplementary material, Table S1). The blue circle identifies the summer phylotypes and red circle is for the winter phylotypes. Halophilic known species are underlined. Same species from different strain with interesting amino acid changes from basic (Lys and K) to polar (Gln, Q and Asn, N) are marked with grey squares (see supplementary Figure S2). The number of redundant phylotypes defined by an identity of 100% is indicated in parenthesis after the accession number. The scale bar represents 0 ± 1 substitutions per aa position. Percentages ≥ 50% of bootstrap are indicated near the nodes. See Section 2 to observe details of the phylogenetic reconstruction done for this tree.

The phylogenetic analysis of the 25 *AprA* Tirez sequences is presented in Figure 5. This analysis included representative species from diverse SRP and SOP taxonomic groups such as Euryarchaeota, Crenarchaeota, Firmicutes, *β*, *γ*, and ∂-proteobacteria (supplementary material, Table S1). The major fraction (16 phylotypes) was affiliated to the SRP. Some of the environmental *AprA* phylotypes were not resolved at genera level, and the result has been discussed for the next taxonomic rank. Cultured and environmental SRP populations were identified as follows: cultured phylotypes (Desulfohalobiaceae, Peptococcaceae, and Desulfobacteraceae) and environmental phylotyeps (Desulfobacteraceae, and Peptococcaceae). One cluster formed by three phylotypes (aps-cw2, -cw4, and -cw5) was closely related to the halotolerant and alkaliphilic *Desulfonatronovibrio hydrogenovorans*. Interestingly, the summer sediment did not reveal the presence of species in the haloadapted Desulfohalobiaceae. Twelve phylotypes obtained from sediment (summer and winter) and enrichments were related to the acetoclastic and nonhalophilic species *Desulfonema magnum*. Two phylotypes (aps-cw6 -es29) were identified as Peptococcaceae. Whilst the phylotype aps-cw6 was conclusively affiliated to *Desulfotomaculum solfataricum* belonging to Firmicutes, the phylotype aps-es29 was not resolved at genera level; however, aps-es29 was allocated basal to the representative Firmicutes taxa used in this study. Actually, the affiliation of phylotype aps-es29 and other SOP Tirez phylotypes could become particularly uncertain given the well-known horizontal APS reductase (Apr) gene transfer (HGT) events between the SRPs from Firmicutes and *δ*-proteobacteria as well as between the SOPs from *β* and *γ*-proteobacteria, respectively (see Figure 5). Both main Apr-HGT events are identified in this work and are in accordance with previous phylogenetic studies [68].

In four of the environmental SOP phylotypes (aps-ew7, -ew8, and -ew13, aps-es28), the assignment of the *aprA* gene fragment could not be conclusive at species level; thus, a detailed function in Tirez's system remains uncertain. The closest clade for three phylotypes was a group of noncultured microorganisms (endosymbionts) in Hydrogenophilaceae in *β*-proteobacteria. The phylotype from summer sediment (aps-es28) remained unidentified at species level, and tree topology helped to designate it as *γ*-proteobacteria. The environmental phylotype aps-ew3 was conclusively affiliated to endosymbionts and close to Thiotrichaceae and Chromatiaceae in *γ*-proteobacteria. Other three phylotypes were derived from enrichment (aps-cw11, -cw12, and -cw13) and resulted with a short distance with the cultured haloalkaliphilic purple bacteria *Thioalkalivibrio* (Ectothiorhodospiraceae) in *γ*-proteobacteria. 

**Figure 6:**
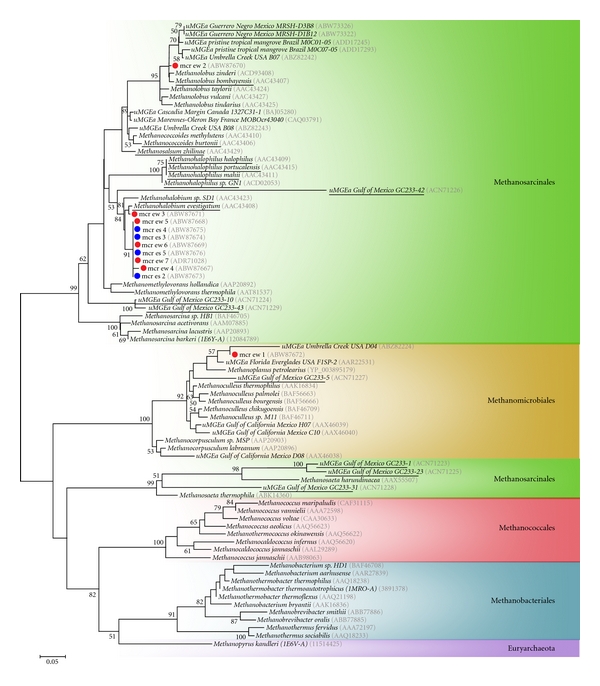
The phylogenetic tree is showing the relationship of *McrA* from Tirez sediments with *McrA *sequences of characterized MA (supplementary material, Table S2). The blue circle identifies the summer phylotypes, and red circle is for the winter phylotypes. Halophilic known species are underlined. The scale bar represents 0 ± 1 substitutions per aa position. Percentages ≥ 50% of bootstrap are indicated near the nodes. See Section 2 to observe details of the phylogenetic reconstruction done for this tree.

The phylogenetic reconstruction of the eleven *McrA* sequences obtained from the anoxic Tirez sediments is shown in Figure 6. This analysis included representative MA species within Methanomicrobiales, Methanosarcinales, Methanococcales, and Methanobacteriales (supplementary material, Table S2). The phylogenetic tree allowed the identification of *McrA* phylotypes belonging to the Methanosarcinaceae and Methanomicrobiaceae. Nine phylotypes were proximate to *Methanohalobium evestigatum *often found in high-salt environments. In the same way, phylotype mcr-ew2 was closely related to *Methanolobus zinderi*. Finally, the phylotype mcr-ew1 closely clustered to the hydrogenotrophic and nonosmoadapted species *Methanoplanus petrolearius*.

### 3.4. Amino Acid Composition, GC content, and Codon Usage Bias in AprA and McrA Phylotypes

The aa composition and GC content in proteins from “salt-in” halotolerant organisms have been related to adaptations to high intracellular concentration in order to favor an osmotic balance within an hypersaline environment [23, 25]. Given that the catalysis of *AprA* and *McrA* enzymes occur in the cytoplasm, we were interested in determine whether Tirez *AprA* and *McrA* sequences show a bias when compared to their halophilic and nonhalophilic homologous counterparts. Thus, we calculated the aa composition from the alignment used to reconstruct the phylogeny in order to estimate the hydrophobic (Gly, Leu, Val, Ile, Phe, Met, Ala, Trp, and Pro), polar (Ser, Thr, Cys, Tyr, Gln, and Asn), basic (His, Arg, and Lys) and acidic (Glu and Asp) contents of the *AprA* and *McrA* enzyme fragments analyzed in this study. Additionally, we used the nucleotide alignment that covers the aa positions selected to reconstruct the phylogeny for each gene marker in order to estimate the general codon bias GC content and the relative synonymous codon usage (RSCU) (see Section 2). For this purpose, we divided the sequence profiles from each gene markers into two data sets the first one is based on salinity adaptation (Tirez, halophilic and nonhalophilic species) and the second one is based on the major taxonomic groups in which the Tirez phylotypes are phylogenetically allocated (*AprA*: Desulfovibrionales, Desulfobacterales, Peptococcales, and Chromatiales; *McrA*: Methanomicrobiales and Methanosarcinales) (supplementary material, Tables S1 and S2, resp.). 

The degree of excess acidic amino acids and dearth of basic amino acids reflects the prevalence of the “salt-in” strategy and the amount of adaptation necessary to cope with the environmental stress. This can be quantified from two estimations: by calculating the surplus of polar and acidic amino acid compared to polar and basic ones (i.e., PAB = [Asx + Glx] − [Arg + Lys]) [57] and by the ratio of the acidic amino acids Glu and Asp to the basic amino acids Lys, His, and Arg (i.e., AB = [Asp + Glu] : [His + Arg + Lys]) [21]. On average, the amino acid composition measurements (Table 1) indicated that *AprA* Tirez phylotypes (PAB = 2.87, AB = 0.62) were from similar to slightly higher in comparison with halophilic (PAB = 2.70, AB = 0.59) and nonhalophilic sequences (PAB = 2.35, AB = 0.62). However, the observed differences in PAB and AB indicators between Tirez phylotypes and halophilic species are out of proportion to argue a “salt-in” signal in Tirez phylotypes given that *AprA* differences are more than ten times less the difference between *Escherichia coli* and *Halobacterium salinarum* or *Halomonas elongata* and *Halobacterium salinarum* [57]. 

**Figure 7:**
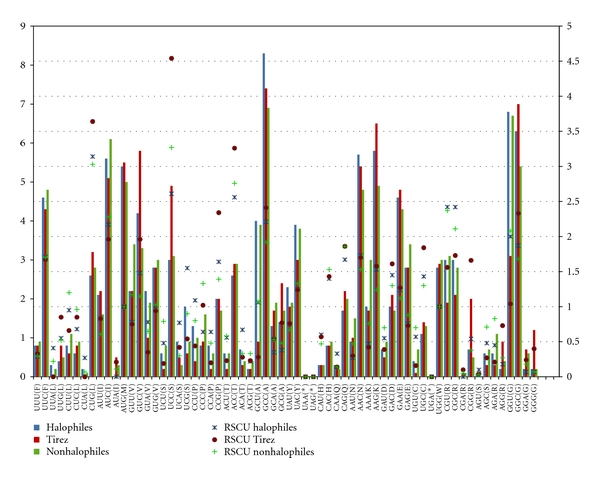
Correspondence analysis of relative synonymous codon usage (RSCU) for *AprA* sequences from halophiles, nonhalophiles, and Tirez phylotypes. The distribution of all codons (including the start and stop codons*) for every amino acid across the three datasets is shown on the *X*-axis. The frequency of each codon (%) is represented with bars on the left *Y*-axis. RSCU values for each codon across the three datasets are represented with differentiated dots on the right *Y*-axis. In the absence of any codon usage bias, the RSCU value would be 1.00. A codon that is used less frequently than expected will have a value of less than 1.00 and *vice versa* for a codon that is used more frequently than expected.

The total GC content of *AprA* Tirez phylotypes, halophiles, and nonhalophiles organisms is 57.60%, 55.70%, and 55.00%, respectively. The GC content of Tirez phylotypes is higher than the reported for *Escherichia coli* (50.3%) but lower than the extreme halotolerant species from the Dead Sea metagenome (62–67%) and *Halobacterium salinarum* (65.7%) (Table 1). A codon usage in *AprA* Tirez phylotypes is consistent with that expected, when corrected for GC composition (Figure 7). In comparison to halophiles and nonhalophiles, *AprA* Tirez phylotypes show a significant overrepresentation of amino residues with a preferential use for a G or C in the third or first position: Val (GUC), Ser (UCC), Gln (CAG), Lys (AAG), Asn (AAC), Asp (GAC), and Glu (GAA). Even though Arg (CGG, AGG), Ala (GCG), and Cys (UGC) are underrepresented amino acids in *AprA* Tirez phylotypes as well as Leu (CUG) and Gly (GGC, GGG) do not show compositional differences when compared with halophiles and nonhalophiles sequences (supplementary material, Figure S1b), all of them show a significant codon usage with GC bias (RSCU > 1.5) (Figure 7). Accordingly, the first, second, and third codon positions of *AprA* Tirez phylotypes have GC percentages of 54.2%, 42.4%, and 76.0%, respectively, and they agree with the GC content values previously reported in some “salt-in” halophiles (Table 1), with high GC content and a third position GC bias [21, 29]. Similar trends on aa composition and GC content can be seen for the *AprA* clades (Table S3 and Figure S1).

A slighter segregation of the *McrA* Tirez phylotypes from the nonhalophilic species is shown in Table 1. Accordingly, the AB indicator for *McrA* phylotypes was slightly lower (1.50) in comparison with the average of halophilic (1.67) and nonhalophilic species (1.75), whilst an opposite trend is shown with the PAB indicator: 16.62 for Tirez, 15.99 for halophiles, and 16.44 for nonhalophiles. In contrast to the *AprA* Tirez phylotypes, the total GC content (47.20%) and the third codon GC bias (46.10%) are significantly lower than the estimated for halophiles and nonhalophilic species (Table 1). The GC content of the first (53.1%) and second (42.4%) codon positions does not change the trend of *McrA* Tirez phylotypes (supplementary material, Figure S3c). Nevertheless, an overrepresentation of amino residues in *McrA* Tirez phylotypes with a preferential codon use (in comparison to nonhalophiles sequences only) can be pointed out for Ile (AUU), Pro (CCA), Ala (GCA), Tyr (UAU), and Asn (AAC, AAU). Even though Lys (AAA), Asp (GAU), Ser (UCC, UCU), and Thr (ACA) are underrepresented amino acids in *McrA* Tirez phylotypes in comparison to halophiles and nonhalophiles sequences (supplementary material, Figure S1b), all of them show a preferential codon usage. Furthermore, it is important to note that the aa composition and GC content trends for *McrA* clade profiles showed a differentiated tendency in contrast to the estimated average from all *McrA* Tirez phylotypes (supplementary material, Table S3). The first, second, and third codon position of Methanomicrobiales present a high GC content values of 52.6% (Tirez 54.1%), 40.0% (Tirez 39.1%), and 73.1% (Tirez 77.4%), respectively. Similarly, the polar and acidic content in Methanomicrobiales (PAB = 17.12 and AB = 1.70) is interestingly higher than the bulk cell protein content reported for *E. coli* (15.85) and close to the haloadaptation threshold of *H. elongata *(17.56) [57].

## 4. Discussion

### 4.1. Identification of Anaerobic Prokaryotes in the Sediment by Functional Gene Approach

SRP and MA are the frequent ecotypes responsible of major biogeochemical processes in sedimentary systems. A functional gene PCR-DGGE approach was applied to identify these anaerobic ecotypes. Regarding the sediment profile and community structure along time and depth, the bands identified in the *aprA* DGGE pattern from environmental samples are in agreement with the presence of black sediments below the evaporite layer observed in summer and winter seasons (Figure 2). This mineral precipitation and the sulfide detected in the sediment (Figure 1(a)) are probably attributable to a dissimilatory sulfate reduction where MA were also detected (Figure 3(c)). The use of a nested PCR implies additional amplification cycles, and, thus, it has been used to increase the visualization sensitivity of species present in low numbers by DGGE [69]. Interestingly, our findings via this approach denote a predominance of the SRP-SOP ecotypes over MA, given that we performed the nested-PCR approach to obtain a positive PCR product of *mcrA *gene fragment, whilst it was not necessary to apply it for the *aprA* gene fragment. Finally, a predominance of SRP-SOP ecotypes in Tirez Lake is in accordance with the high values of sulfate registered on the sediment.

After the clustering analysis of sedimentary populations represented in the *aprA* DGGE pattern, the changes are better explained by a seasonal disturbance in accordance with the ephemeral lagoon. It is suggestible that population resilience is mainly regulated by changes in salinity because the main nodes indicate a partition into dry and flooded patterns (Figure 3(c)); note that salinity fluctuates from 6% (w/v) during winter to 35% (w/v) during spring. However, the strong temperature oscillation can be also associated with salinity over community composition. Additionally, the *P* values (>0.05) indicate that the partition winter/summer is not significant enough to describe well-differentiated communities since flooded node and dry node are more clustered than expected by chance. 

Interestingly, most of the SOP, SRP, and MA phylotypes obtained in this work were related to environmental sequences described from alkaliphilic or thalassic hypersaline systems [6, 20]. However, few data is available from athalassic systems [70]. In SRP were detected phylotypes (aps-cw4, -cw-5, and -cw2) from *Desulfonatronovibrio hydrogenovorans,* a lithoheterotrophic, halotolerant (grows in a salinity range of 1–12% NaCl), and alkaliphilic sulfate respirer. Surprisingly, *D. hydrogenovorans* does not grow at pH of 7 and the highest pH of Tirez is below 8.0. Desulfohalobiaceae species are commonly adapted to high osmolarity due to the anabolic metabolism of compatible solute synthesis and dependent on the use of lactate and hydrogen as electron donors [4]. *Desulfohalobium retbaense* is considered the neutrophilic and thalassic counterpart of *D. hydrogenovorans,* but it was not detected in Tirez.

Gram-Positive *Desulfotomaculum solfataricum* (aps-cw6) was detected in enrichments. Another phylotype, aps-es29, is also a member of Peptococcaceae, but it could not be assigned to a specific genus. These phylotypes did not cluster with *Desulfotomaculum halophilum* sequences, which tolerates up to 12% NaCl [71]. However, a previous study reports *Desulfotomaculum* isolates in a salt pan [72]. Sulfate-reducing bacteria in Peptococcaceae perform oxidation from a broad spectrum of electron donors such as lactate [73]. Compatible solutes in Peptococcaceae have not been characterized; however, the theoretical energy yield, for example, in medium supplied with lactate is Δ*G*°′ = −160 kJ/mol, would give enough energy for the osmoprotectant synthesis or transport as, for example*, Desulfovibrio vulgaris*; *D. vulgaris *is trophically analog to *Desulfotomaculum *species. *D. vulgaris* synthesizes sugars such as trehalose or accumulates amino acids such as glycine betaine and proline as compatible solutes as response to under salt stress. Stress response in *D. vulgaris* is based on genes with homologous in diverse and distant species such as *Bacillus subtilis* [74]; thus, the finding of Peptococcaceae in Tirez, under analog bioenergetic constraints, could be explained in the terms of the “salt-out” strategy (see Section 4). 

The presence of *Methanohalobium evestigatum* and *Methanolobus zinderi *in the sulfate-rich and anoxic sediment is easily sustained by functional arguments, even in summer samples, because their metabolism requires methylated substrates; thus, it is noncompetitive with SRP. *M. evestigatum* and *M. zinderi* are theoretically productive in bioenergetic terms [75], enough to exhibit compatible solute synthesis [76]. *Methanolobus zinderi* was isolated from an estuary and grows at the higher rate and tolerates upper levels of divalent cations (Mg^2+^) in comparison with monovalent Na^+^ [77]. This characteristic is remarkable because *M. zinderi* could be adequate to Tirez given that divalent cation Ca^2+^ dominate over monovalents in the sediment (Figure 1(e)). On the other hand, the increase of ammonium (NH_4_^+^ 4–6 *μ*M) at 10–15 cm depth and the decrease of Eh across the sediment profile (Figure 1) suggest the development of strict anaerobic and methylotrophic MA metabolisms [78]. 

None of the genera detected in both seasons clustered with acetoclastic MA. The absence of acetoclastic MA in hypersaline systems has been widely accepted as a consequence of the low Gibbs free energy dissipated from acetate as substrate [4]. However, acetoclastic MA activity was reported in Napoli mud volcano brines with 4.0 M chloride, where the Cl : SO_4_ ratio is 200 times higher than the observed in Tirez [79]. In Tirez, the absence of acetoclastic MA is probably explained by substrate outcompetition, because the sulfate-reducing conditions prevail due to the high abundance of sulfate in Tirez and to the putative adaptation of acetoclastic SRP such as *Desulfonema magnum* to the extreme sediment. 

The sulfur-oxidizing populations have been frequently described in extreme hypersaline systems. Some of the phylotypes from environmental and enrichment culturing were affiliated to endosymbionts; its potential ecological role in the sediment is supported by the view that the sulfur cycle has been described in marine oligochaetes, where endosymbionts identified as proteobacterial microorganisms participate as sulfur oxidizers [80]. Therefore, it is plausible that the free-living and nonisolated relative populations in Tirez sediment have an analogous metabolic role. Three phylotypes from winter sediment and enrichments were affiliated to the chemoautotrophic genus *Thioalkalivibrio* and sulfur oxidizing endosymbionts in *β*/*γ*-proteobacteria clade (Figure 5). These anaerobic ecotypes are expected to be found in the extremely saline sediment as much as the H_2_S is present (Figure 1(a)); in turn, H_2_S would be oxidized anaerobically by these purple bacteria given that low Eh and partial O_2_ pressure were observed in the sampling site (Figure 1). The discrepancy in the finding of* Thioalkalivibrio* is due to its narrow range of optimal pH (9.5–10.0), the fact that species in *Thioalkalivibrio* are true alkaliphilic and are well adapted to athalassic soda lakes, that is, dominated by monovalent cations [81], and considering that other sulfur oxidizing and halophilic SOP species such as *Thiomicrospira halophila* or *Hallochromatium* spp. [82] were not detected and probably better adapted to neutral Tirez saltern. Unfortunately, the SOP Tirez phylotype from summer sediment was not identified at species level. 

It has been argued that hypersaline environments are inappropriate for the biological development of anaerobic acetate oxidation as a consequence of the low negative balance of the standard Δ*G* yielded by this dissimilatory metabolism and due to the high maintenance energy needed for the synthesis/accumulation of compatible solute under high osmotic conditions [4]. However, at high sulfate concentrations, *Desulfonema magnum* populations were unequivocally detected in the evaportitic sediment and winter sediment samples (environmental and derived from enrichment culturing) at 0–15 cm depth under an extreme salinity stress of 35% salts. This acetate-oxidizing Desulfobacteraceae has not been described in hypersaline systems and was the most abundant phylotype identified in Tirez lagoon. *D. magnum* has an optimal salinity about 2.5% NaCl and has been described in marine microbial mats [83]. Previous studies have shown that Desulfobacteraceae are present in thalassic hypersaline basins [70] and athalassic soda lakes [6]. This is a notable finding for the understanding of carbon cycle in extreme hypersaline ecotypes because under extreme conditions there is a decline in organic matter remineralization; thereby, organisms encoding the corresponding *aprA* gene probably face the salinity changes. Halophilic species from Desulfobacterales have not been isolated; *Desulfobacter halotolerans* is member of Desulfobacterales but has an optimum growth with only of 1-2% NaCl [84]. Nevertheless, very little is known about the mechanisms involved in energy conservation that allow acetoclastic SRP organisms to survive in extreme saline conditions. The haloadaptation mechanism “salt-in” osmoadaptation has been suggested for Desulfobacteraceae ecotypes identified in soda lakes to compensate saline stress [6]. Possibly, *Desulfonema,* being an acetoclastic SR, exerts additional energy conserving mechanisms (as in the case of MA and acetogenic bacteria) consisting in extra transference of electrons from membrane complexes dependent on H^+^ or Na^+^ pumping. Such process is likely to occur in the acetoclastic Desulfobacteraceae *Desulfobacterium autrotrophicum* whose conservation mechanism of chemiosmotic energy is analogous to that in homoacetogenic bacteria [85].

A *mcrA* phylotype from the hydrogenotrophic *Methanoplanus petrolearius *was detected in the surface DGGE profile from winter sediment at 0–5 cm depth (Figure 4), when salt content in the saltern is averaged at 6% w/v. This organism has a maximum tolerance at 5% and an optimal growth at 1–3% NaCl [86]. It is feasible that the *M. petrolearius* salt tolerance determines its absence in summer samples and is correlated with the low energy yielded by the methanogenic pathway based on H_2_ and formate as electron donors. MT activity based on these substrates has a low theoretical energy yielded (Δ*G*°′). Therefore, it is plausible that *M. petrolearius* is less abundant than methylotrophic MA. *Methanoplanus *clones, which have been reported in thalassic hypersaline sediments but at 2.2 M Cl^−^ and sulfate below the detection limit [87].

### 4.2. Halotolerant Strategies in Tirez Lagoon

In order to adjust to lower water activities of the environment and the resulting decrease in cytoplasmic water, microorganisms must accumulate intracellular ions or organic solutes to reestablish the turgor pressure and preserve enzyme activity [27, 88]. “Salt-in” halophiles are adapted to hypersaline environments by a mechanism that involves at least equimolar extracellular and intracellular salt concentrations by a selective influx of potassium ions into the cytoplasm. The “salt-in” strategy favors solubility and is energetically efficient, but unfolds proteins at high concentration [24]. As a consequence, this halotolerant strategy requires that the entire intracellular machinery, that is, proteins, nucleic acids,s and their specific interactions with one another, must be adapted to high salt intracellular levels. The adaptations generally include an increase of the acidic nature of intracellular proteins and/or an increment of genomic CG content and a GC-bias at the codon usage level. Nevertheless, Paul et al. [25] demonstrated common genomic and proteomic trends in halophiles that transcend the boundary of phylogenetic relationship and the genomic GC content of the species. Accordingly, it has been suggested that distantly lineages adopted “salt-in” strategy independently by convergent evolution given its radical nature [27]. 

All previous studies have estimated average trends of amino acid composition and GC content from selected sequences or enzymes in marine aerobic populations [21, 57] or from completely sequenced genomes obtained from diverse aerobic environments [22, 25]. Even though “salt-in” strategy was recently proposed to explain the finding of the resilience *Desulfobacteraceae* at hypersaline and alkaline lakes [6], this salt-adaptation strategy has been neither reported in species of the SRP-SOP nor in MA; in part, given the absence of complete sequenced genomes and sequenced 16s RNAs from uncultured species. Therefore, we consider it useful to use *AprA* and *McrA* markers to test “salt-in” signals. An intuitive justification would be to expect a naturally biased selection for *AprA* and *McrA* enzymes given their frequent or higher expression levels in the cytoplasm (in comparison to other encoded genes at the genome) in order to cope with their ecological and metabolic role on anaerobic and hypersaline sediments.

Our results cannot be conclusive regarding the halotolerant strategies carried out by Tirez phylotypes, until a large sequence data set can be achieved for these organisms. Nevertheless, the amino acid composition, GC content, and preferential codon usage trends exhibited by the *AprA* marker from Tirez phylotypes suggest a plausible “salt-in” signal when compared to halophiles and non-haphiles. The increase in negatively charged (Asp and Glu) and polar (Ser, Asn, and Gln) residues in *AprA* Tirez phylotypes can be explained by a codon usage with GC bias at the third position. The overrepresentation of these amino acid residues and their preferential codon usage are consistent with reports on “salt-in” adaptation [22, 25]. Similarly, a higher frequency of Val in *AprA* Tirez phylotypes compared to nonhalophiles and halophiles supports the observation of Madern et al. [24] and Paul et al. [25], but disagree with earlier propositions on underrepresentation of all strong hydrophobic residues in halophiles [89]. We also report a slight decrement of the basic residue Arg in *AprA* Tirez phylotypes. The role of Arg in haloadaptation is quite controversial; its increment in halophilic species can be expected by mutational bias [25] given that five of the six codons have a bias towards GC nucleotides; however, Arg has been also reported in a consistent decrement in specific haloadapted species [29, 57]. Even though the slight increment of Lys observed in *AprA* Tirez phylotypes contradicts all previous propositions on underrepresentation of the most important basic residues in all “salt-in” halophiles [22, 25], it has been recently suggested that dipeptides like Val-Lys significantly contribute to the halostability in proteins [90]. 

As described on results, the mcr-ew1 Tirez phylotype allocated in Methanomicrobiales shows an interestingly phylogenetic tendency to use amino acids, not initially biased by GC content or codon usage, that could be involved in a weak-moderate “salt-in” strategy. For example, slight increments of the polar residues Asn, Ser, and Tyr, the negatively charged residues Asp and Glu, and the hydrophobic residues Ala, Ile, and Pro in *McrA* Methanomicrobiales phylotypes are in agreement with salt-in signals previously reported [57]. Charged amino acids prevent charged ions from attaching to proteins and thus they have a significant role in stabilizing proteins against salty conditions and keeping water molecules around these proteins [25, 50]. Similar to *AprA* Tirez phylotypes, we observed a decrement of Arg and an increment of Lys (supplementary material, Table S3). The remaining *McrA* Tirez phylotypes do not exhibit a clear tendency about expected aa composition, GC content, and codon usage bias to carry out “salt-in” haloadaptation. These phylotypes could compensate high salt extracellular concentrations through mechanisms independent of amino acid composition and GC content and that do not compromise the enzymatic activity [91]. The “salt-out” strategy requires the accumulation of specific small-molecular-weight compounds (i.e., compatible solutes or osmolytes) into the cytoplasm. Thereby, “salt-out” signal can be expected on the *McrA* Tirez phylotypes close clustered at *Methanohalobium evestigatum* and *Methanohalobium sp.* species belonging to the Methanosarcinales. This observation is in agreement with the compatible solute characterization described for this clade [92]. It is also well know that *M. evestigatum* uses methylated compounds such as methylamine and methanol to generate methane. These methylated substrates not only provide more energy to *M. evestigatum *than the use of others substrates for anabolic reactions, including the synthesis of compatible solutes, but also allow a tolerance up to 29.2% of NaCl [93]. In “salt-out” strategy, little or no adjustment is required to intracellular macromolecules; in fact, the compatible solutes often act as more general stress protectants as well as just osmoprotectants [27].

Furthermore, halophiles do not live at constant salt concentrations; but in many natural settings they are exposed to changing salinities due to evaporation or rain, and thus also the intracellular conditions change considerably [23]. Accordingly, enzyme activity on “salt-in” halophilic strategy will depend not only on the nature and concentration of the salt, but also on extensive genetic alterations as a prerequisite for adaptation to a saline intracellular environment [24, 27]. Tajima's neutrality test [94] for the *AprA* and *McrA* enzyme fragments (used in this study) shows that both gene markers are evolving under positive selection (*D*_*AprA*_ = 3.13 and  *D*_*AprA*_ = 2.96) (supplementary material, Table S4). This means that key functional enzymes of anaerobic microorganisms on Tirez lagoon could undergone extensive genetic alterations that, if they help the organism to cope and adapt with a saline intracellular environment, could be clearly differentiated and fast fixed on the populations. Two clear examples of this flexible genetic alterations and selective fixation can be seen on *AprA* sequences of same species but that were obtained from different strains: *Thermodesulforhabdus norvegica* DSM 9990 (EF442952.1) [95]; AF418159.1, [68] and *Archaeoglobus fulgidus* DSM 4304 (PDB: 1JNR-A) [16]; PDB: 2FJA-A, [48], which show interesting amino acid changes from basic (Lys and K) to polar (Gln, Q and Asn, N) residues (see Figure 5 and supplementary Figure S2).

In spite of the considerable diversity in nucleotide content and amino acid composition of the *AprA* and *McrA* enzyme fragments involved in all analyses, it can be seen a crucial conservation of catalytic sites (Arg-R^*α*265^-Trp-W^*α*234^ in* AprA* and Phe-F^*α*330^-Tyr-Y^*α*333^-Phe-F^*α*443^-Gly-G^*α*445^ in *McrA*) as well as of cofactor and nucleotide binding sites in both gene markers (Figures S1 and S2 for *AprA* and *McrA* aa alignments, resp.). As previously reported for *McrA* [50], the same conservative trend holds true for most of the surrounding residues of the *AprA* and *McrA* catalytic sites. Probably, the amino acid conservation and/or the structural localization of these catalytic regions on *AprA* and *McrA *gene markers underestimate the general trend composition of “salt-in” adaptation from moderately to high halotolerant organisms in Tirez lagoon. In fact, it is not possible to figure out at the moment if the diversity, weakness, or absence of amino acid, GC content, and codon usage patterns reported for Tirez phylotypes in this study are a consequence of a minor and biased coverage of their not completely sequenced genomes or if these inconclusive trends are true salt-in signals or a consequence of the use of complementary salt-adaptation strategies in bioenergetically constrained species, given that Tirez phylotypes have a clear anaerobic mode of life on highly saline and sulfate sediments.

Accordingly, we do not discard the presence of mixed types of osmoadaptation in *AprA* and *McrA* Tirez phylotypes, where K^+^ accumulates to high levels (“salt-in”) along with neutral and negatively charged organic solutes (“salt-out”), as previously reported for many slightly and moderately halophilic methanogens [96]. For example, *Methanohalophilus portucalensis* grows in 2.0 M NaCl and its intracellular concentration of K^+^ is 0.76 M, indicating that concentration of intracellular K^+^ need not be the same as that of extracellular Na^+^. Presumably, *M. portucalensis* uses three zwitterions and other osmolytes to balance osmotic pressure [92, 96]. Likewise, K^+^ plays an important role in the response of *Methanococcus thermolithotrophicus* to hyperosmotic (increased NaCl) or hypoosmotic (decreased NaCl) shock. At the beginning of higher NaCl extracellular concentration, *M. thermolithotrophicus* internalizes K^+^ until reach a new steady-state intracellular concentration; then, synthesis and accumulation of L-*α*-glutamate occur. The K^+^-*α*-glutamate pair functions as a temporary osmolyte whilst the nonmetabolizable zwitterion (Ne-acetyl-b-lysine) is synthesized and accumulated by *M. thermolithotrophicus* exclusively in response to high salt concentrations [96, 97].

### 4.3. Implications of Anaerobic Diversity for Tirez Biogeochemistry

The characterization of SRP, SOP, and MA diversity in Tirez lagoon contributes to the knowledge of anaerobic diversity of microorganisms in athalassohaline systems and has inferences on the survival and adaptation of life under steep salt gradients. A characterization of the anaerobic diversity in Tirez lagoon is a first step to explain functional issues such as why not all anaerobic dissimilatory pathways occur optimally in extreme biotopes and whether an anaerobic way of life faces higher energetic constraints in hypersaline systems in terms of salt composition [4]. Any quantitative interpretation can be inferred because PCR-DGGE fingerprint is an inconclusive source of information and fluorescence *in situ* hybridization (FISH), parallel experiments designed specifically to quantify ∂-proteobacteria and methanogen populations along the sediment profile (winter and sediment), failed to yield any positive result (data not shown). 

The structure and activity of hydrogenotrophic methanogenesis and acetoclastic sulfidogenesis under thalassic hypersaline systems have been extensively studied [98, 99]. But, it should be kept in mind that Tirez sediment is a sulfate-rich system with a peculiar salt composition, considering that in the evaporitic period minerals such as gypsum (CaSO_4_·2H_2_O), epsomite (MgSO_4_ ·7H_2_O), and hexahydrite (MgSO_4_·6H_2_O) are deposited and dominate over halite (NaCl) [60], and most importantly, the sulfate has a relevant role in anaerobic systems as electron acceptor. Thus, present results might be of importance for the understanding of acetate mineralization as a key process for carbon cycling in extreme environments. In Tirez sulfate-rich sediment, among all the detected phylotypes, *Desulfonema magnum *and *Methanoplanus petrolearius* are the ecotypes of major interest due to energetic constraints; therefore, these ecotypes constitute a probable signal of haloadaptation in anaerobic populations. 

Although it was possible to characterize several anaerobic prokaryotes involved in distinctive metabolic lineages across the Tirez sediment, DGGE and phylogenetic analyses revealed a poor SRP, SOP, and MA phylotype composition; probably underestimated in comparison with other extreme systems [100]. Nevertheless, extant conditions in Tirez, as well as in other hypersaline environments, enable the persistence of low energetic anaerobic metabolic capabilities such as the *Halanaerobiales *fermenting bacteria (manuscript in prep.), which use a well-adapted fermentation of organic compounds to produce CO_2_/H_2_ and volatile fatty acids (VFA) such as acetate by the use of the “salt-in” strategy [101]. 

Typically, the carbon cycle in halophilic communities implicates low rates of carbon mineralization to CO_2_ which explains the accumulation of acetate at salt saturation levels [102]. In addition to H_2_ and acetate, methylated compounds as fermentation products of compatible solutes can be mineralized by MA [78, 103]. The perspective for the nitrogen cycle is different in Tirez, its completion is predictable given that it shares the characteristics of other hypersaline systems, where methylotrophic MA contribute to nitrogen mineralization [103]. About the sulfur cycle, the sulfate-reducing microorganisms were identified in the sulfate-rich sediment and represent probable suppliers of sulfide for sulfur-oxidizing populations. This understanding is useful to infer possible biological processes in analogous systems such as Europa because the ocean present in the satellite is rich in sulfates and divalent cations and probably it is also in anoxic state [31, 104].

## 5. Conclusion

Extensive phylogenetic and physiological characterizations of thalassic and alkaline anaerobic biotopes have been reported. Phylogenetic studies have been traditionally determined by physiological characterization of marine species, and the records of anaerobic phylotypes in hypersaline systems are dominated by thalassic species. Tirez lagoon has *sabkha* properties thus, it is a brine of interest to analyze strong spectra in salinity. Also, Tirez lagoon is characterized by a low chloride/sulfate ratio; this is remarkable considering that sulfate serves as terminal electron acceptor in the marine systems; however, few biological descriptions have been made when this anion is abundant under hypersaline conditions. Using the PCR-DGGE fingerprint technique for the functional adenosine-5′-phosphosulfate (*aprA*) and the methyl coenzyme M reductase (*mcrA*) gene markers, we have confirmed the occurrence of hydrogenotrophic methanogenic and acetoclastic sulfate-reducing organisms in Tirez sediment. Despite the steep osmotic change along the year in the lagoon, changes in composition of PCR-DGGE dendrogram reflected weak differences on winter-summer community structure.

The persistence of Desulfobacteraceae phylotypes in summer sediment as well as the finding of Methanomicrobiales at the hypersaline and sulfate-rich sediment is remarkable (hydrogenotrophic MA are outcompeted by SRP in high concentrations of sulfate). Probably, these ecotypes are energetically constrained and, unfortunately, our findings on amino acid and nucleotide compositions cannot be currently conclusive regarding the halotolerant strategies carried out by Tirez phylotypes until a large sequence data set can be achieved for these uncultured, anaerobic and bioenergetically constrained organisms. Nevertheless, it looks like *AprA* gene marker could be a useful “salt-in” indicator for different environmental (e.g., marine versus sedimentary) samples, not only because its amino acid overrepresentation and codon usage bias well correlate with those found in halophiles but also because *AprA* gene marker could exhibit a preferential use of amino acid (e.g., Val and Lys) on sediments in contrast to those found in marine and aerobic environments. Similarly, *McrA* gene marker shows an unexpected amino acid and nucleotide composition with nonclear “salt-in” signals exhibited. However, we speculate that the diverse and not conclusive salt-in signals in these ecotypes (perhaps due to the absence of complete sequenced *McrA* genes) could reflect that whereas protective osmolytes “salt-out” can be produced by MA Tirez populations in response to salt stress, probably also a weak “salt-in” strategy may contribute to adaptation of osmotic stress on sedimentary MA Tirez populations.

An extended understanding for acetoclastic sulfate reducing activity under high osmolarity conditions is needed in order to elucidate mechanisms that are involved in the biological carbon mineralization. On the long term, the findings of this work will provide valuable information to determine habitable conditions of Europa, the most interesting moon of Jupiter for the Astrobiology field, as an anoxic and hypersaline environment.

##  Authors' Contribution

L. Montoya, I. Lozada-Chávez, I. Marín and R. Amils conceived the study. L. Montoya, I. Marín, R. Amils and N. Rodriguez were involved in the fieldwork. L. Montoya performed the experimental work. I. Lozada-Chávez performed the sequence analysis. L. Montoya and I. Lozada-Chávez performed the analysis and interpretation of data, and wrote the paper. All authors read, improved, and approved the final paper.

## Supplementary Material

Supplementary Tables:
Table S1: Description of the *aprA* gene sequences used to reconstruct the phylogeny.Table S2: Description of the *mcrA* gene sequences used to reconstruct the phylogeny.Table S3: The amino acid composition and G*+*C content of *McrA* and *AprA* Tirez phylotypes and their corresponding phylogenetic clades.Table S4: Results from Tajima's Neutrality Test for *AprA* and *McrA* gene sequences.
Supplementary Figures:
Figure S1: Alignment of 66 amino acid sequences with the corresponding fragment to the *AprA*, N-terminal domain. (A) Description of the catalytic sites for the *AprA*. (B) Relative amino acid composition of the *AprA* catalytic region. (C) Nucleotide codon composition for the *aprA* gene.Figure S2: Alignment of 56 amino acid sequences with the corresponding fragment to the *McrA*, C-terminal domain. (A) Description of the catalytic sites for the *McrA*. (B) Relative amino acid composition of the *McrA* catalytic region. (C) Nucleotide codon position for the *mcrA* gene.Figure S3: Correspondence analysis of Relative Synonymous Codon Usage (RSCU) for *McrA* sequences from halophiles, non-halophiles and Tirez phylotypes.

## Figures and Tables

**Table 1:** The amino acid composition and G + C content of Tirez McrA and AprA sequences, their halophilic and nonhalophilic homologous counterparts, metagenomes, and reference strains.

	Acid Asx + Glx	Acid − Basic^6^ (Asx + Glx) − (Arg + Lys)	Acid : Basic^7^ (Asp + Glu) : (His + Arg + Lys)	Lys	Asp : Lys	Arg	G + C % in total sequence^8^	G + C % in third codon position^8^
*AprA *								

Tirez^1^	15.90 ± 2.24	2.87 ± 2.54	0.62 ± 0.13	6.90 ± 1.40	0.35 ± 0.22	6.14 ± 0.90	57.60 ± 4.56	76.00 ± 9.99
Halophilic species^2^	14.90 ± 2.74	2.70 ± 3.02	0.59 ± 0.18	6.20 ± 0.90	0.30 ± 0.09	6.02 ± 0.59	55.70 ± 7.56	66.50 ± 20.70
Nonhalophilic species^2^	15.00 ± 1.95	2.35 ± 2.13	0.62 ± 0.12	6.30 ± 1.10	0.35 ± 0.11	6.34 ± 0.68	55.00 ± 6.94	65.50 ± 19.02

*mcrA*								

Tirez^1^	22.07± 0.23	16.62 ± 0.24	1.50 ± 0.00	3.38 ± 0.62	2.04 ± 0.37	2.07 ± 0.40	47.20 ± 3.40	46.10 ± 11.70
Halophilic species^3^	21.09 ± 0.52	15.99 ± 0.76	1.67 ± 0.10	3.54 ± 0.29	2.08 ± 1.83	1.56 ± 0.00	51.50 ± 5.20	57.00 ± 12.60
Nonhalophilic species^3, 4^	21.75 ± 1.22	16.44 ± 1.63	1.75 ± 0.35	3.65 ± 0.99	2.08 ± 0.80	1.67 ± 0.3	50.30 ±7.20	56.30 ± 21.30

Metagenomic^7^								

Dead Sea	n.d.	n.d.	1.46	n.d.	n.d.	n.d.	62–67	n.d.

Reference strains^5^								

*Halobacterium salinarum*	31.80	25.36	n.d.	2.34 ± 0.04	n.d.	4.10 ± 0.12	65.7	n.d.
*Halomonas elongata*	25.98	17.56	n.d.	3.7	n.d.	5.25	n.d.	n.d.
*Escherichia coli*	26.04	15.85	n.d.	6.03 ± 0.14	n.d.	4.16 ± 0.02	50.3–50.9	n.d.

^1^Average composition from amino acid sequences derived from this study.

^2^Average composition from amino acid sequences listed in supplementary material Table S1.

^3^Average composition from amino acid sequences listed in supplementary material Table S2.

^4^Thermophilic species were not included.

^5^Amino acid composition of the bulk protein content in type species cultures [57].

^6^PAB: amino acid proportions according to [57].

^7^AB: amino proportions according to Rhodes et al.[21].

^8^GC content percentage is calculated as GC% = (G + C/G + C + A + T) ∗ 100.
