# Supplementary material for: The Sulfate-Rich and Extreme Saline Sediment of the Ephemeral Tirez Lagoon: A Biotope for Acetoclastic Sulfate-Reducing Bacteria and Hydrogenotrophic Methanogenic Archaea
Source: Int J Microbiol. 2011 Sep 11;2011:753758. doi: 10.1155/2011/753758 (PMC3170894; doi:10.1155/2011/753758)
Supplement: Supplementary file 1 — Supplementary Tables: Table S1: Description of the aprA gene sequences used to reconstruct the phylogeny.Table S2: Description of the mcrA gene sequences used to reconstruct the phylogeny.Table S3: The amino acid composition and G+C content of McrA and AprA Tirez phylotypes and their corresponding phylogenetic clades.Table S4: Results from Tajima's Neutrality Test for AprA and McrA gene sequences. Supplementary Figures: Figure S1: Alignment of 66 amino acid sequences with the corresponding fragment to the AprA, N-terminal domain. (A) Description of the catalytic sites for the AprA. (B) Relative amino acid composition of the AprA catalytic region. (C) Nucleotide codon composition for the aprA gene.Figure S2: Alignment of 56 amino acid sequences with the corresponding fragment to the McrA, C-terminal domain. (A) Description of the catalytic sites for the McrA. (B) Relative amino acid composition of the McrA catalytic region. (C) Nucleotide codon position for the mcrA gene.Figure S3: Correspondence analysis of Relative Synonymous Codon Usage (RSCU) for McrA sequences from halophiles, non-halophiles and Tirez phylotypes. [file 753758.f1.pdf]

Supplementary material for:

The sulfate rich and extreme saline sediment of the ephemeral Tirez lagoon: a biotope for acetoclastic sulfate reducing bacteria and hydrogenotrophic methanogenic archaea

Lilia Montoya, Irma Lozada-Chávez, Ricardo Amils, Nuria Rodriguez, Irma Marín

CONTENT

		Page
**Table**		
S1	Description of the *aprA* gene sequences used to reconstruct the phylogeny	2
S2	Description of the *mcrA* gene sequences used to reconstruct the phylogeny	4
S3	The amino acid composition and G+C content of *McrA* and *AprA* Tirez phylotypes and their corresponding phylogenetic clades.	7
S4	Results from Tajima's Neutrality Test for *AprA and McrA* gene sequences	7
**Figure**		
S1	Alignment of 66 amino acid sequences with the corresponding fragment to the *AprA*, N-terminal domain	8
a	Description of the catalytic sites for the *AprA*	10
b	Relative amino acid composition of the *AprA* catalytic region	11
c	Nucleotide codon composition for the *aprA* gene	12
S2	Alignment of 56 amino acid sequences with the corresponding fragment to the *McrA*, C-terminal domain	13
a	Description of the catalytic sites for the *McrA*	15
b	Relative amino acid composition of the *McrA* catalytic region	16
c	Nucleotide codon position for the *mcrA* gene	17
S3	Correspondence analysis of Relative Synonymous Codon Usage (RSCU) for *McrA* sequences from halophiles, non-halophiles and Tirez phylotypes	18
S4	Diagram of the target sites for primer pairs within *Mcr* gene	19

Table S1. Description of the *aprA* gene sequences used to reconstruct the phylogeny and to analyze the amino acid composition.

#	Phylotype name (phylogenetic tree)	Nucleotide GenBank Accession GI	Protein Genbank Accession GI	Protein NCBI Acession ID	UniProt / Swissprot ID	Reference
Bacteria, Proteobacteria						
**(β) Betaproteobacteria**						
1	*Thiobacillus denitrificans* ATCC 25259	NC_007404	74318300	YP_316040	Q3SGL5	Copeland et al., 2006
2	*Thiobacillus plumbophilus* DSM 6690	EF641917.1	157804760	ABV80021	A8JZE1	Meyer and Kuever, 2007
3	*Thiobacillus thioparus* DSM 505	EF641920.1	157804769	ABV80027	A8JZE7	Meyer and Kuever, 2007
**(γ) Gammaproteobacteria**						
4	*Thioalkalivibrio sp.* K90mix	NC_013889	289207252	YP_003459318	D3SC63	Lucas et al., 2010
5	*Thiohalocapsa halophila* DSM 6210	EF641932.1	157804804	ABV80050	A8JZH0	Meyer and Kuever, 2007
6	*Thiolamprovum pedioforme* DSM 3802	EF641941.1	157804831	ABV80068	A8JZI8	Meyer and Kuever, 2007
7	*Chromatium okenii* 6210	EF641935.1	157804813	ABV80056	A8JZH6	Meyer and Kuever, 2007
8	*Halochromatium glycolicum* DSM 11080	EF641934.1	157804810	ABV80054	A8JZH4	Meyer and Kuever, 2007
9	*Thiorhodococcus minor* DSM 11518	EF641950.2	209916496	ABV80084	A8JZK4	Meyer and Kuever, 2007
10	*Rhabdochromatium marinum* DSM 5261	EF641947.1	157804846	ABV80078	A8JZJ8	Meyer and Kuever, 2007
11	*Thiorhodovibrio winogradskyi* DSM 6702	EF641946.1	157804843	ABV80076	A8JZJ6	Meyer and Kuever, 2007
12	*Allochromatium warmingii* DSM 173	EF641931.1	157804801	ABV80048	A8JZG8	Meyer and Kuever, 2007
13	*Thermochromatium tepidum* DSM 3771	EF641936.1	157804816	ABV80058	A8JZH8	Meyer and Kuever, 2007
14	*Thiococcus pfennigii* DSM 226	EF641942.1	157804834	ABV80070	A8JZJ0	Meyer and Kuever, 2007
15	*Thiocapsa rosea* DSM 235	EF641961.1	157804886	ABV80104	A8JZM4	Meyer and Kuever, 2007
16	*Thiocystis violacea* DSM 214	EF641949.1	157804852	ABV80082	A8JZK2	Meyer and Kuever, 2007
17	*Thiodictyon bacillosum* DSM 234	EF641915.1	157804754	ABV80017	A8JZD7	Meyer and Kuever, 2007
18	*Thiothrix nivea* DSM 5205	EF641919.1	157804766	ABV80025	A8JZE5	Meyer and Kuever, 2007
19	*Thiothrix sp.* 12730	EF641918.1	157804763	ABV80023	A8JZE3	Meyer and Kuever, 2007
20	*Lamprocystis purpurea* DSM 4197	EF641909.1	157804736	ABV80005	A8JZC5	Meyer and Kuever, 2007
21	endosymbiont of *Inanidrilus leukodermatus*	EF641926.1	157804787	ABV80039	A8JZF9	Meyer and Kuever, 2007
**Unkown Beta or Gamma proteobacteria**						
22	endosymbiont of *Oligobrachia haakonmosbiensis*	AM883194.1	198033775	CAP03144	B5D6E9	Losekann et al., 2008
23	endosymbiont of *Sclerolinum contortum*	AM883195.1	198033777	CAP03145	B5D6F0	Losekann et al., 2008
24	endosymbiont of *Lucinoma aff. kazani*	AM236338.1	113431892	CAJ85653	Q0KG00	Duperron et al., 2007
**(δ) Deltaproteobacteria**						
25	*Syntrophobacter fumaroxidans* MPOB	NC_008554.1	116748490	YP_845177	A0LH40	Copeland et al., 2006
26	*Thermodesulforhabdus norvegica* DSM 9990	EF442952.1	151302340	ABR92558	A6YCY8	Meyer and Kuever, 2007
27	*Thermodesulforhabdus norvegica* DSM 9990	AF418159.1	18034251	AAL57426	Q8VRT9	Friedrich, 2002
28	*Desulfacinum hydrothermale* DSM 13146	AF418148.1	18034228	AAL57415	Q8VRV0	Friedrich, 2002
29	*Desulforhabdus sp.* DDT	EF442951.1	151302338	ABR92557	A6YCY7	Meyer and Kuever, 2007
30	*Desulfarculus baarsii* DSM 2075	EF442946.1	151302323	ABR92547	A6ZJS7	Meyer and Kuever, 2007
31	*Desulfomonile tiedjei* DSM 6799	EF442948.1	151302329	ABR92551	A6YCY1	Meyer and Kuever, 2007
32	*Desulfonema magnum* str. Montpellier DSM 2077	EF442926.1	151302263	ABR92507	A6YCU3	Meyer and Kuever, 2007
33	*Desulfonema limicola* str. Jadebusen DSM 2076	EF442925.1	151302260	ABR92505	A6YCU1	Meyer and Kuever, 2007
34	*Desulfonema ishimotonii* DSM 9680	AF418135.1	18034203	AAL57402	Q8VRW3	Friedrich, 2002
35	*Desulfobacterium indolicum* DSM 3383	EF442911.1	151302218	ABR92477	A6YCR7	Meyer and Kuever, 2007
36	*Desulfobacterium sp.* PM4	EF442913.1	151302224	ABR92481	A6YCS1	Meyer and Kuever, 2007
37	*Desulfobacterium autotrophicum* HRM2	NC_012108.1	224367567	YP_002601730	C0QHK8	Meyer and Kuever, 2007
38	*Desulfobacterium zeppelinii* DSM 9120	EF442917.1	151302236	ABR92489	A6ZJR3	Meyer and Kuever, 2007
39	*Desulfatibacillum alkenivorans* AK-01	NC_011768.1	218779417	YP_002430735	B8FAH1	Lucas et al., 2008
40	*Desulfosarcina variabilis* str. Montpellier DSM 2060	EF442928.1	151302269	ABR92511	A6YCU7	Meyer and Kuever, 2007
41	*Desulfococcus sp.* DSM 8541	EF442923.1	151302254	ABR92501	A6YCT7	Meyer and Kuever, 2007
42	*Desulfobacula toluolica* Tol2 DSM 7467	EF442919.1	151302242	ABR92493	A6YCT0	Meyer and Kuever, 2007
43	*Desulfospira joergensenii* DSM 10085	EF442929.1	151302272	ABR92513	A6YCU9	Meyer and Kuever, 2007
44	*Desulfobacter sp.* DSM 2057	EF442909.1	151302212	ABR92473	A6YCR3	Meyer and Kuever, 2007
45	*Desulfocella halophila* DSM 11763	AF418117.1	18034167	AAL57384	Q8VRX9	Friedrich, 2002
46	*Desulforhopalus vacuolatus* DSM 9700	EF442940.1	151302305	ABR92535	A6YCX0	Meyer and Kuever, 2007

47	*Desulfobulbus marinus* DSM 2058	EF442934.1	151302287	ABR92523	A6ZJR7	Meyer and Kuever, 2007
48	*Desulforegula conservatrix* DSM 13527	EF442927.1	151302266	ABR92509	A6YCU5	Meyer and Kuever, 2007
49	*Desulfonatronovibrio hydrogenovorans* DSM 9292	EF442902.1	151302192	ABR92460	A6YCQ1	Meyer and Kuever, 2007
50	*Desulfomicrobium baculatum* DSM 4028	EF442899.1	151302183	ABR92454	A6YCP5	Meyer and Kuever, 2007
51	*Desulfocaldus sp.* Hobo	EF442898.1	151302180	ABR92452	A6YCP3	Meyer and Kuever, 2007
52	*Desulfothermus naphthae* DSM 13418	EF442901.1	151302189	ABR92458	A6YCP9	Meyer and Kuever, 2007
53	*Desulfohalobium retbaense* DSM 5692	EF442900.1	151302186	ABR92456	A6YCP7	Meyer and Kuever, 2007
54	*Desulfovibrio piger* ATCC 29098 *****	AF418129.1	18034191	AAL57396	Q8VRW7	Friedrich, 2002
55	*Desulfovibrio sulfodismutans* DSM 3969	EF442897.1	151302177	ABR92450	A6YCP1	Meyer and Kuever, 2007
56	*Desulfonauticus autotrophicus* DSM 4206	EF442904.1	151302198	ABR92464	A6ZJQ7	Meyer and Kuever, 2007
57	*Desulfonatronum lacustre* DSM 10312	AF418137.1	18034207	AAL57404	Q8VRW1	Friedrich, 2002
58	*Desulfovibrio sp.* DSM 9953	EF442888.1	151302150	ABR92432	A6YCM3	Meyer and Kuever, 2007
59	*Desulfovibrio desulfuricans subsp. desulfuricans* str. G20	NC_007519.1	78356157	YP_387606	Q313I5	Copeland et al., 2005
60	*Desulfovibrio gigas* (PDB: 3GYX-A)		281307059			Chiang et al., 2009
Bacteria, Thermodesulfobacteria						
Thermodesulfobacteriaceae						
61	*Thermodesulfatator indicus*	EF442880.1	151302126	ABR92416	A6YCK7	Meyer and Kuever, 2007
62	*Thermodesulfobacterium hveragerdense* DSM 12571	EF442879.1	151302123	ABR92414	A6YCK5	Meyer and Kuever, 2007
Bacteria, Nitrospirae						
Nitrospiraceae						
63	*Thermodesulfovibrio yellowstonii* DSM 11347	EF442881.1	151302129	ABR92418	A6YCK9	Meyer and Kuever, 2007
64	*Thermacetogenium phaeum* DSM 12270	EF442974.1	151302401	ABR92597	A6YD27	Meyer and Kuever, 2007
Bacteria, Firmicutes						
Clostridia						
65	*Desulfotomaculum sp.* DSM 8775	EF442968.1	151302384	ABR92586	A6YD16	Meyer and Kuever, 2007
66	*Desulfotomaculum thermobenzoicum subsp. thermobenzoicum* DSM 6193	EF442970.1	151302390	ABR92590	A6YD20	Meyer and Kuever, 2007
67	*Desulfotomaculum thermoacetoxidans* DSM 5813	EF442969.1	151302387	ABR92588	A6YD18	Meyer and Kuever, 2007
68	*Desulfotomaculum solfataricum* DSM 14956	EF442963.1	151302369	ABR92576	A6YD06	Meyer and Kuever, 2007
69	*Desulfotomaculum luciae* DSM 12396	EF442960.1	151302361	ABR92571	A6YD01	Meyer and Kuever, 2007
70	*Desulfotomaculum halophilum* DSM 11559	AF418167.1	18034267	AAL57434	Q8VRT2	Friedrich, 2002
71	*Desulfotomaculum alkaliphilum* DSM 12257	AF418154.1	18034241	AAL57421	Q8VRU4	Friedrich, 2002
72	*Desulfotomaculum acetoxidans* DSM 771	AF418153.1	18034239	AAL57420	Q8VRU5	Friedrich, 2002
Bacteria, Creanarchaeota						
Thermoprotei						
73	*Pyrobaculum aerophilum* str. IM2	NC_003364.1	18313433	NP_560100	Q8ZUX4	Fitz-Gibbon et al., 2002
Bacteria, Euryarchaeota						
Archaeoglobi						
74	*Archaeoglobus fulgidus* DSM 4304 (PDB: 1JNR-A)		20150473		O28603	Roth et al., 2000<br>Fritz et al., 2002
75	*Archaeoglobus fulgidus* DSM 4304 (PDB: 2FJA-A)	NC_000917.1	11499260	NP_070498	O28603	Klenk et al., 1997<br>Schiffer et al., 2006
76	*Archaeoglobus veneficus* DSM 11195	EF442877.1	151302117	ABR92410	A6YCK1	Meyer and Kuever, 2007
77	*Archaeoglobus profundus* DSM 5631	EF442876.1	151302114	ABR92408	A6YCJ9	Meyer and Kuever, 2007
***Bacteria*, Several *Phyla* (this work)**						
78	Phylotype aps cw 1	EU722715	193160529	ACF15331	B3V9Y4	aps1
79	Phylotype aps cw 2	EU722726	193160551	ACF15342	B3V9Z5	aps2
80	Phylotype aps cw 3	EU722716	193160531	ACF15332	B3V9Y5	aps3
81	Phylotype aps cw 4	EU722725	193160549	ACF15341	B3V9Z4	aps4
82	Phylotype aps cw 5	EU722717	193160533	ACF15333	B3V9Y6	aps5
83	Phylotype aps cw 6	EU722718	193160535	ACF15334	B3V9Y7	aps6
84	Phylotype aps cw 8	EU722719	193160537	ACF15335	B3V9Y8	aps8
85	Phylotype aps cw 11	EU722721	193160541	ACF15337	B3V9Z0	aps11
86	Phylotype aps cw 12	EU722722	193160543	ACF15338	B3V9Z1	aps12

87	Phylotype aps cw 13	EU722723	193160545	ACF15339	B3V9Z2	aps13
88	Phylotype aps cw 20	HM466937	313585805	ADR71020	E5FFS6	aps20
89	Phylotype aps ew 3	EU722728	193160555	ACF15344	B3V9Z7	apsam3
90	Phylotype aps ew 4	HM466938	313585807	ADR71021	E5FFS7	apsam4
91	Phylotype aps ew 7	HM466939	313585809	ADR71022	E5FFS8	apsam7
92	Phylotype aps ew 8	HM466940	313585811	ADR71023	E5FFS9	apsam8
93	Phylotype aps ew 13	EU722729	193160557	ACF15345	B3V9Z8	apsam13
94	Phylotype aps ew17	HM466943	313585813	ADR71024	E5FFT0	apsam17
95	Phylotype aps es 22	HM466944	313585815	ADR71025	E5FFT1	apsam22
96	Phylotype aps es 23	HM466945	313585817	ADR71026	E5FFT2	apsam23
97	Phylotype aps es 24	EU722730	193160559	ACF15346	B3V9Z9	apsam24
98	Phylotype aps es 25	EU722731	193160561	ACF15347	B3VA00	apsam25
99	Phylotype aps es 28	HM466946	313585819	ADR71027	E5FFT3	apsam28
100	Phylotype aps es 29	EU722732	193160563	ACF15348	B3VA01	apsam29

***** Recent sequence allocation from Desulfomonas pigra

Table S2. Description of the *mcrA* gene sequences used to reconstruct the phylogeny to analyze the amino acid composition.

#	Phylotype name (phylogenetic tree)		Nucleotide GenBank Accession ID	Protein GenBank Acession ID	UniProt / Swissprot ID	Reference
Archaea; Euryarchaeota; Methanobacteria; Methanobacteriales						
Methanobacterium						
1	*Methanobacterium aarhuse* H2-LR	AY386125	38946222	AAR27839	Q6TWD8	Shlimon et al., 2004
2	*Methanobacterium bryantii* DSM 863	AF313806	13259181	AAK16836	Q9C4K5	Lueders et al., 2001
3	*Methanobacterium sp.* HD-1	AB288269	125215261	BAF46708	A2V7N3	Shimizu et al., 2006
Methanobrevibacter						
4	*Methanobrevibacter oralis* DSM 7256	DQ251045	82492007	ABB77885	Q1PS59	Vianna et al., 2006
5	*Methanobrevibacter smithii* ATCC 35061 str. DSM 861	DQ251046	82492009	ABB77886	Q1PS58	Vianna et al., 2006
Methanothermobacter						
6	*Methanothermobacter thermoflexus* DSM 7268	AY303950	33520725	AAQ21198	Q7ZA88	Lausten et al., 2003
7	*Methanothermobacter thermophilus* DSM 6529	AY289752	33415457	AAQ18238	Q7ZA91	Lausten et al., 2003
8	*Methanothermobacter thermoautotrophicus* ***** (PDB:1MRO-A)		3891378			Ermler et al., 1997
Methanothermus						
9	*Methanothermus sociabilis* DSM 3496	AY289747	33415447	AAQ18233	Q7ZA96	Lausten et al., 2003
10	*Methanothermus fervidus*	J03375	149800	AAA72197	P12971	Weil et al., 1988
Archaea; Euryarchaeota; Methanococci; Methanococcales						
Methanocaldococcus						
11	*Methanocaldococcus infernus* SL47	AY354031	34017058	AAQ56620	Q6V254	Nercessian et al., 2005
12	*Methanocaldococcus jannaschii* DSM 2661	AF414040	16798075	AAL29289	Q58256	Luton et al., 2002
Methanococcus						
13	*Methanococcus aeolicus* DSM 4304	AY354034	34017064	AAQ56623	Q6V252	Nercessian et al., 2005
14	*Methanococcus jannaschii* DSM 2661	L77117	1590865	AAB98063	Q60391	Bult et al., 1996
15	*Methanococcus maripaludis* S2	BX950229	45047997	CAF31115	Q6LWZ5	Hendrickson et al., 2004
16	*Methanococcus vannielii*	M16893	150059	AAA72598	P07961	Cram et al., 1987
17	*Methanococcus voltae* PS	X07793	44729	CAA30633	P11559	Klein, 1988
Methanothermococcus						
18	*Methanothermococcus okinawensis* DSM 14208	AY354033	34017062	AAQ56622	Q6V253	Nercessian et al., 2005
Archaea; Euryarchaeota; Methanomicrobia; Methanomicrobiales						
Methanocorpusculum						
19	*Methanocorpusculum labreanum*	AY260441	30230509	AAP20896	Q877F6	Simankova et al., 2003
20	*Methanocorpusculum sp.* MSP	AY260448	30230523	AAP20903	Q877E9	Simankova et al., 2003
21	Uncultured methanogenic archaeon (uMGEa) Gulf of California Mexico clone D08	AY837763	61398332	AAX46038	Q49KC4	Dhillon et al., 2005
Methanoculleus						

22	*Methanoculleus chikugoensis* JCM 10825	AB288270	125215264	BAF46709	A2V7N4	Shimizu et al., 2006
23	*Methanoculleus thermophilus* DSM 2624	AF313804	13259177	AAK16834	Q9C4K7	Lueders et al., 2001
24	*Methanoculleus sp.* M11	AB288285	125215331	BAF46711	A2V7N6	Shimizu et al., 2006
25	*Methanoculleus palmolei* INSULZ DSM 4273	AB300784	145370902	BAF56663	A4PJ19	Watanabe et al., 2009
26	*Methanoculleus bourgensis* MS2 DSM 3045	AB300787	145370912	BAF56666	A4PJ22	Watanabe et al., 2009
27	*Methanoplanus petrolearius* DSM 11571	NC_014507	307354128	YP_003895179	E1RJD5	Lucas et al., 2010<br>Brambilla et al., 2010
28	Uncultured methanogenic archaeon (uMGEa)<br>Umbrella Creek USA clone D04AUGControl	EU302029	167541428	ABZ82224	B0Z9B2	Edmonds et al., 2008
29	Uncultured methanogenic archaeon (uMGEa)<br>Florida Everglades USA clone MCR-F1SP-2	AY458406	38503475	AAR22531	Q6JIA4	Castro et al., 2004
30	Uncultured methanogenic archaeon (uMGEa)<br>Gulf of Mexico clone GC 233mcr5	FJ754031	224969354	ACN71227	C0LR95	Joye et al., 2009
31	Uncultured methanogenic archaeon (uMGEa)<br>Gulf of California Mexico clone H07	AY837764	61398334	AAX46039	Q49KC3	Dhillon et al., 2005
32	Uncultured methanogenic archaeon (uMGEa)<br>Gulf of California Mexico clone C10	AY837765	61398336	AAX46040	Q49KC2	Dhillon et al., 2005
Archaea; Euryarchaeota; Methanomicrobia; Methanosarcinales						
Methanohalobium						
33	*Methanohalobium evestigatum*	U22236	799185	AAC43408	Q49114	Springer et al., 1995
34	*Methanohalobium sp.* SD-1	U22256	799215	AAC43423	Q50387	Springer et al., 1995
35	Uncultured methanogenic archaeon (uMGEa)<br>Gulf of Mexico clone GC 233mcr42	FJ754030	224969352	ACN71226	C0LR94	Joye et al., 2009
Methanohalophilus						
36	*Methanohalophilus halophilus* Z-7982	U22259	799187	AAC43409	Q49535	Springer et al., 1995
37	*Methanohalophilus mahii*	U22237	799191	AAC43411	Q50215	Springer et al., 1995
38	*Methanohalophilus portucalensis*	U22238	799199	AAC43415	Q50273	Springer et al., 1995
39	*Methanohalophilus sp.* GN1	EU585974	187236232	ACD02053	B2Z3B5	Orphan et al., 2008
Methanolobus						
40	*Methanolobus tindarius*	U22244	799219	AAC43425	Q50536	Springer et al., 1995
41	*Methanolobus vulcani*	U22245	799223	AAC43427	Q50828	Springer et al., 1995
42	*Methanolobus taylorii*	U22243	799217	AAC43424	Q50535	Springer et al., 1995
43	*Methanolobus bombayensis* B-1	U22257	799183	AAC43407	Q48922	Springer et al., 1995
44	*Methanolobus zinderi*	EU715818	189396511	ACD93408	B3GQ22	Doerfert et al., 2009
45	Uncultured methanogenic archaeon (uMGEa)<br>Umbrella Creek USA clone AUGCONTROLB07	EU302047	167541464	ABZ82242	B0Z9D0	Nolling et al., 1996
46	Uncultured methanogenic archaeon (uMGEa)<br>pristine tropical mangrove Brazil clone M0C07_05	GU395780	289719421	ADD17293	D3Y3W7	Taketani et al., 2010
47	Uncultured methanogenic archaeon (uMGEa)<br>pristine tropical mangrove Brazil clone M0C01_05	GU395722	289719316	ADD17245	D3Y3R9	Taketani et al., 2010
48	Uncultured methanogenic archaeon (uMGEa)<br>Guerrero Negro Mexico clone MRSH-D1B12	EU147790	158537760	ABW73322	A8UQB4	Potter et al., 2009
49	Uncultured methanogenic archaeon (uMGEa)<br>Guerrero Negro Mexico clone MRSH-D3B8	EU147794	158537768	ABW73326	A8UQC8	Potter et al., 2009
Methanococcoides						
50	*Methanococcoides burtonii* DSM 6242	U22234	799181	AAC43406	Q48921	Springer et al., 1995
51	*Methanococcoides methylutens*	U22235	799189	AAC43410	Q50214	Springer et al., 1995
52	Uncultured methanogenic archaeon (uMGEa)<br>Umbrella Creek USA clone AUGCONTROLB08	EU302048	167541466	ABZ82243	B0Z9D1	Edmonds et al., 2008
53	Uncultured methanogenic archaeon (uMGEa)<br>Marennes-Oleron Bay France clone MOBOcr43040	AM942090	219918963	CAQ03791	B7ZG30	Roussel et al., 2009
54	Uncultured methanogenic archaeon (uMGEa)<br>Cascadia Margin Canada clone 1327C31-1	AB525700	294488845	BAJ05280	D4QE49	Yoshioka et al., 2009
Methanomethylovorans						
55	*Methanomethylovorans hollandica* ZB	AY260437	30230501	AAP20892	Q877G0	Simankova et al., 2003

56	*Methanomethylovorans thermophila* L2FAW	AY672820	50818137	AAT81537	Q6B9V5	Jiang et al., 2005
Methanosalsum						
57	*Methanosalsum zhilinae* **	U22252	799227	AAC43429	Q50908	Springer et al., 1995
Methanosaeta						
58	*Methanosaeta harundinacea*	AY970349	61743608	AAX55507	Q58F45	Ma et al., 2006
59	*Methanosaeta thermophila* PT	CP000477		ABK14360	A0B6N6	Copeland et al., 2006
60	Uncultured methanogenic archaeon (uMGEa) Gulf of Mexico clone GC 233mcr1	FJ754027	224969346	ACN71223	C0LR91	Joye et al., 2009
61	Uncultured methanogenic archaeon (uMGEa) Gulf of Mexico clone GC 233mcr23	FJ754029	224969350	ACN71225	C0LR93	Joye et al., 2009
62	Uncultured methanogenic archaeon (uMGEa) Gulf of Mexico clone GC 425mcr31	FJ754032	224969356	ACN71228	C0LR96	Joye et al., 2009
Methanosarcina						
63	*Methanosarcina acetivorans* C2A	AE010299	19918691	AAM07885	Q8THH1	Galagan et al., 2002
64	*Methanosarcina lacustris* MM	AY260438	30230503	AAP20893	Q877F9	Simankova et al., 2003
65	*Methanosarcina sp*. HB-1	AB288266	125215254	BAF46705	A2V7N0	Shimizu et al., 2006
66	*Methanosarcina barkeri* (PDB:1E6Y-A)		12084789			Grabarse et al., 2000
**Uncultured**						
67	Uncultured methanogenic archaeon (uMGEa) Gulf of Mexico clone GC 233mcr10	FJ754028	224969348	ACN71224	C0LR92	Joye et al., 2009
68	Uncultured methanogenic archaeon (uMGEa) Gulf of Mexico clone GC 425mcr43	FJ754033	224969358	ACN71229	C0LR97	Joye et al., 2009
Archaea; Euryarchaeota; Methanopyri; Methanopyrales						
Methanopyrus						
69	*Methanopyrus kandlery* (PDB:1E6V-A)		11514425			Rospert et al., 1991 Grabarse et al., 2000
***Archaea; Euryarchaeota* (this work)**						
70	Phylotype mcr ew 4	EU091355	159032574	ABW87667	B4XT53	1A_1
71	Phylotype mcr ew 5	EU091356	159032576	ABW87668	B4XT54	1A_2
72	Phylotype mcr ew 6	EU091357	159032578	ABW87669	B4XT54	1A_3
73	Phylotype mcr ew 2	EU091358	159032580	ABW87670	B4XT56	1A_4
74	Phylotype mcr ew 3	EU091359	159032582	ABW87671	B4XT57	3A_1
75	Phylotype mcr ew 1	EU091360	159032584	ABW87672	B4XT58	3A_2
76	Phylotype mcr ew 7	HM466948	313585821	ADR71028	E5FFT4	3A_6
77	Phylotype mcr es 2	EU091361	159032586	ABW87673	B4XT54	18A_1
78	Phylotype mcr es 3	EU091362	159032588	ABW87674	B4XT54	18A_2
79	Phylotype mcr es 4	EU091363	159032590	ABW87675	B4XT54	18A_3
80	Phylotype mcr es 5	EU091364	159032592	ABW87676	B4XT62	18A_4

* *Metanothermobacter thermoautotrophicus*, new name for *Methanobacterium thermoautotrophicum* (Wasserfallen et al., 2000) and recent sequence allocation by *Methanothermobacter marburgensis* str. Marburg.
** *Methanosalsum zhilinae*, recent sequence allocation by *Methanohalophilus zhilinaeae.*

Table S3. The amino acid composition and G+C content of *McrA* and *AprA* Tirez phylotypes and their corresponding phylogenetic clades.

	Acid<br>Asx + Glx	Acid – Basic[1]<br>(Asx + Glx) – (Arg + Lys)	Acid : Basic[2]<br>(Asp + Glu) : (His + Arg + Lys)	Lys	Asp : Lys	Arg	G+C % in total sequence[3]	G+C % in third codon position[3]
AprA clades								
Desulfovibrionales	16.80 ± 1.08	5.29 ± 1.65	0.66 ± 0.07	5.20 ± 0.80	0.33 ± 0.05	6.30 ± 0.20	55.50 ± 6.80	68.40 ± 18.50
Desulfovibrionales (Tirez)	14.95 ± 1.07	2.50 ± 1.50	0.65 ± 0.03	5.50 ± 0.50	0.32 ± 0.07	7.00 ± 0.80	60.60 ± 4.10	81.60 ± 9.20
Desulfobacterales	17.01 ± 0.77	4.00 ± 1.17	0.71 ± 0.05	6.40 ± 0.70	0.34 ± 0.11	6.60 ± 0.60	49.80 ± 5.00	52.90 ± 15.50
Desulfobacterales (Tirez)	17.00 ± 0.85	3.30 ± 1.44	0.62 ± 0.05	7.80 ± 0.40	0.27 ± 0.11	6.00 ± 0.90	53.80 ± 2.90	68.20 ± 8.40
Peptococcales	13.73 ± 0.37	1.74 ± 0.29	0.75 ± 0.09	5.70 ± 0.00	0.45 ± 0.05	6.30 ± 0.40	55.40 ± 6.60	65.50 ± 1.97
Peptococcales (Tirez)	15.75 ± 0.25	5.15 ± 1.50	0.74 ± 0.01	4.70 ± 0.30	0.45 ± 0.07	5.90 ± 0.90	62.90 ± 1.30	88.00 ± 0.60
Chromatiales-SOP	14.31 ± 2.06	1.22 ± 1.71	0.51 ± 0.13	7.30 ± 0.30	0.30 ± 0.09	5.80 ± 0.50	59.70 ± 5.20	77.10 ± 14.90
Chromatiales-SOP (Tirez)	14.83 ± 3.32	1.91 ± 3.72	0.57 ± 0.20	6.80 ± 1.50	0.38 ± 0.34	6.10 ± 0.80	59.90 ± 3.54	79.90 ± 7.70
McrA clades								
Methanomicrobiales	22.70 ± 0.64	17.12 ± 1.19	1.70 ± 0.21	4.01 ± 0.75	2.01 ± 0.50	1.57 ± 0.20	55.20 ± 4.80	73.1 ± 14.6
Methanomicrobiales (Tirez)	22.73	16.67	1.50	4.55	1.50	1.52	56.9	77.40
Methanosarcinales	20.95 ± 1.21	15.48 ± 1.12	1.58 ± 0.21	3.93 ± 0.80	1.88 ± 0.67	1.54 ± 0.13	50.50 ± 0.20	54.80 ± 12.10
Methanosarcinales (Tirez)	22.00 ± 0.07	16.62 ± 0.25	1.50 ± 0.00	3.26 ± 0.51	2.12 ± 0.22	2.12 ± 0.32	46.30 ± 0.94	42.80 ± 3.86

1) PAB: Amino acid proportions according to Oren (2002).
2) AB: Amino proportions according to Rhodes (2010, 2010).
3) GC content percentage is calculated as: GC%= (G+C/G+C+A+T) * 100

Table S4. Results from Tajima's Neutrality Test for *AprA and McrA* gene sequences. *Abbreviations*: *m* = number of sequences, *S* = Number of segregating sites, *p*s = *S*/*m*, Θ = *p*s/a_1, π = nucleotide diversity, and *D* is the Tajima test statistic. The Tajima's test of neutrality (Tajima, 1989) compares the number of segregating sites per site with the nucleotide diversity (a site is considered segregating if, in a comparison of m sequences, there are two or more nucleotides at that site; nucleotide diversity is defined as the average number of nucleotide differences per site between two sequences). If all the alleles are selectively neutral, then the product 4Nv (where N is the effective population size and v is the mutation rate per site) can be estimated in two ways, and the difference in the estimate obtained provides an indication of non-neutral evolution. When D is equal or near to 0 then, the sequence(s) are neutrally evolving, whilst D > 0 indicates a positive selection and D < 0 indicates negative or purifying selection of the sequence(s). The analysis involved the same amino acid alignments used for the phylogenetic reconstruction (described below), and it was conducted in MEGA5 (Tamura et al., 2007).

Gene marker	***m***	***S***	***ps***	***Θ***	***π***	***D***
AprA	100	117	0.936000	0.180787	0.351669	3.127999
McrA	80	101	0.765152	0.154483	0.290585	2.967687

Figure S1. Alignment of 100 amino acid sequences with the corresponding fragment to the *AprA*, N-terminal domain. *AprA* from 2 to 261 amino acid positions has been characterized in *Archaeoglobus fulgidus* in the reduced state (FAD_{red}-APS, PDB ID: 1JNR) (Fritz et al., 2002) and in the oxidized state (FAD_{ox}-APS, PDB ID: 2FJA) (Schiffer et al., 2006) as well as in *Desulfovibrio gigas* (PDB ID: 3GYX) (Chiang et al., 2009). This alignment[1] includes two of the nine functional active sites (marked with black background) of the Apr_alpha_N domain: Arg-$R^{\alpha 265}$ and Trp-$W^{\alpha 234}$ (Klein et al.) reported by Schiffer et al., (2006). Essential catalytic amino acids for cofactor and nucleotide binding sites are marked with grey backgrounds as reported from PDB and UniProt databases. Same species from different strain with interesting amino acid changes from basic (Lys, K) to polar (Gln, Q and Asn, N) are marked with blue backgrounds. 122 from 137 positions were used for the phylogenetic reconstruction.

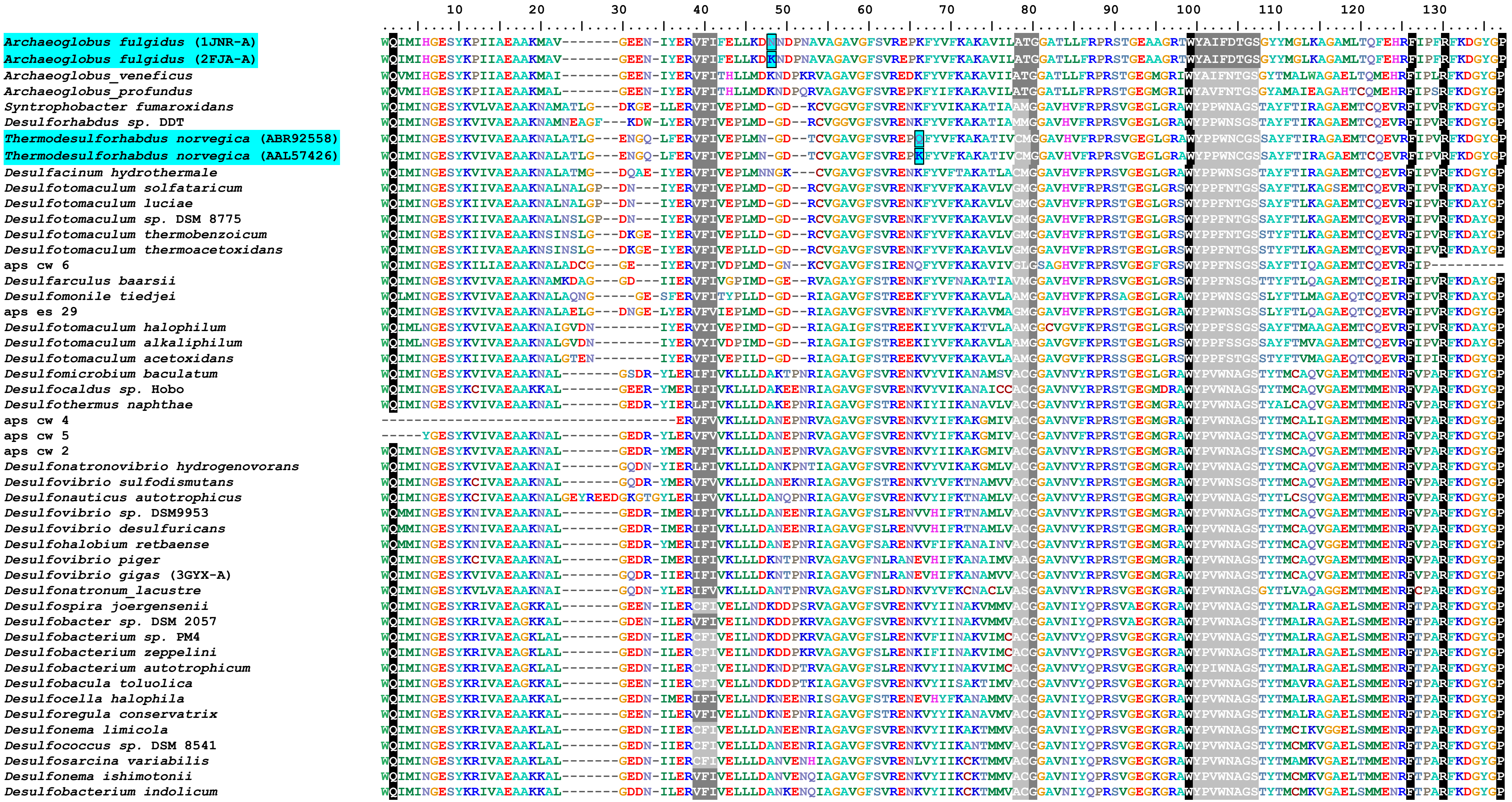

[1] Same species from different strain with interesting amino acid changes from basic (Lys, K) to polar (Gln, Q and Asn, N) are marked with blue color.

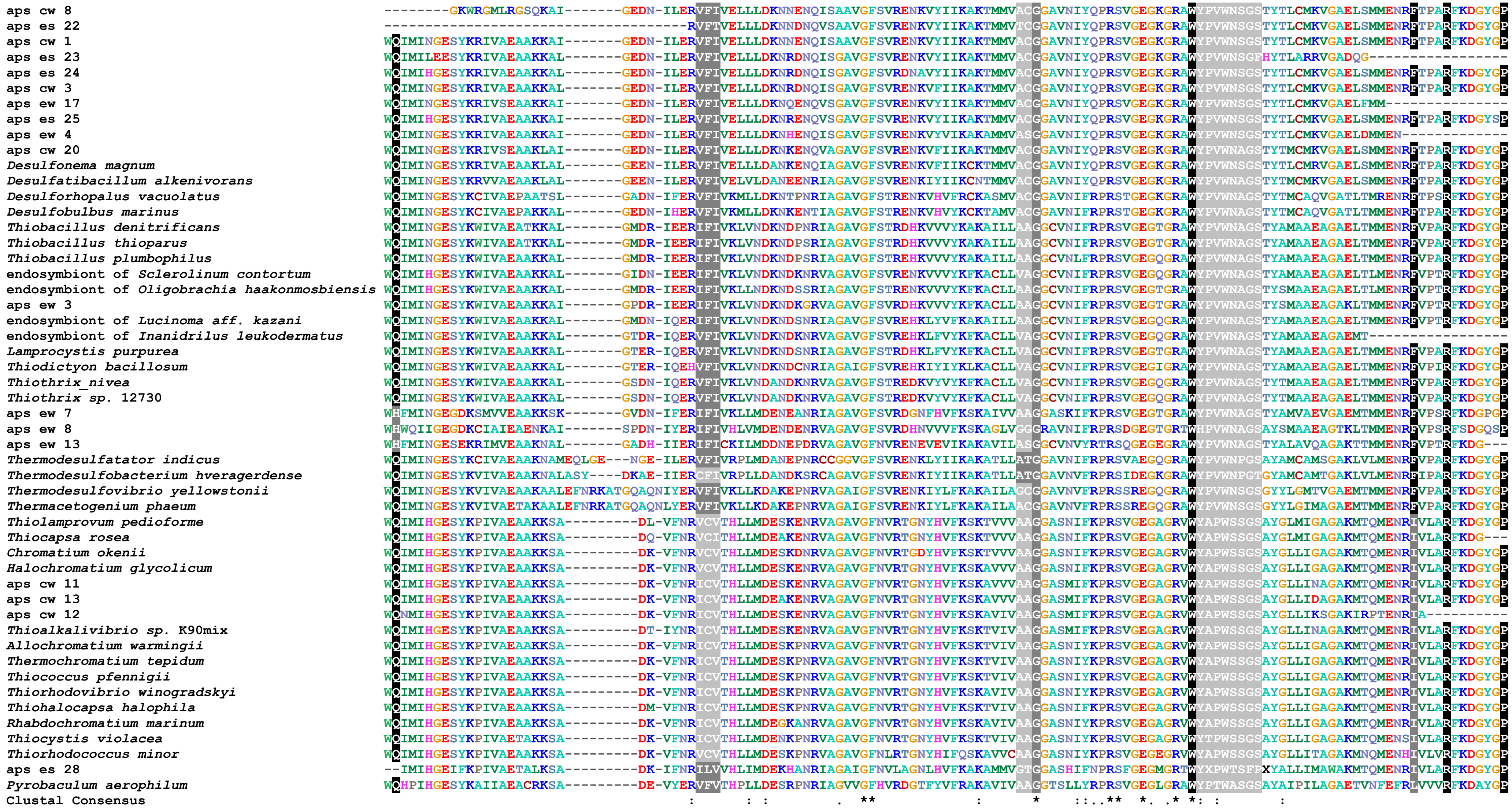
aps cw 8
aps es 22
aps cw 1
aps es 23
aps es 24
aps cw 3
aps ew 17
aps es 25
aps ew 4
aps cw 20
Desulfonema magnum
Desulfatibacillum alkenivorans
Desulforhopalus vacuolatus
Desulfobulbus marinus
Thiobacillus denitrificans
Thiobacillus thioparus
Thiobacillus plumbophilus
endosymbiont of Sclerolinum contortum
endosymbiont of Oligobrachia haakonmosbiensis
aps ew 3
endosymbiont of Lucinoma aff. kazani
endosymbiont of Inanidrilus leukodermatus
Lamprocystis purpurea
Thiodictyon bacillosum
Thiothrix_nivea
Thiothrix sp. 12730
aps ew 7
aps ew 8
aps ew 13
Thermodesulfatator indicus
Thermodesulfobacterium hveragerdense
Thermodesulfovibrio yellowstonii
Thermacetogenium phaeum
Thiolamprovum pedioforme
Thiocapsa rosea
Chromatium okenii
Halochromatium glycolicum
aps cw 11
aps cw 13
aps cw 12
Thioalkalivibrio sp. K90mix
Allochromatium warmingii
Thermochromatium tepidum
Thiococcus pfennigii
Thiorhodovibrio winogradskyi
Thiohalocapsa halophila
Rhabdochromatium marinum
Thiocystis violacea
Thiorhodococcus minor
aps es 28
Pyrobaculum aerophilum
Clustal Consensus

Figure S1. a) Description of the catalytic sites for *AprA*.

Figure of the *Apr* crystal and its active site region, modified from Schiffer (2006).

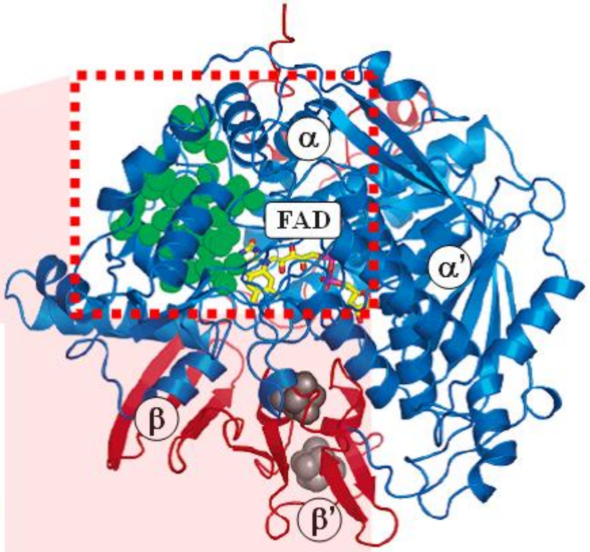

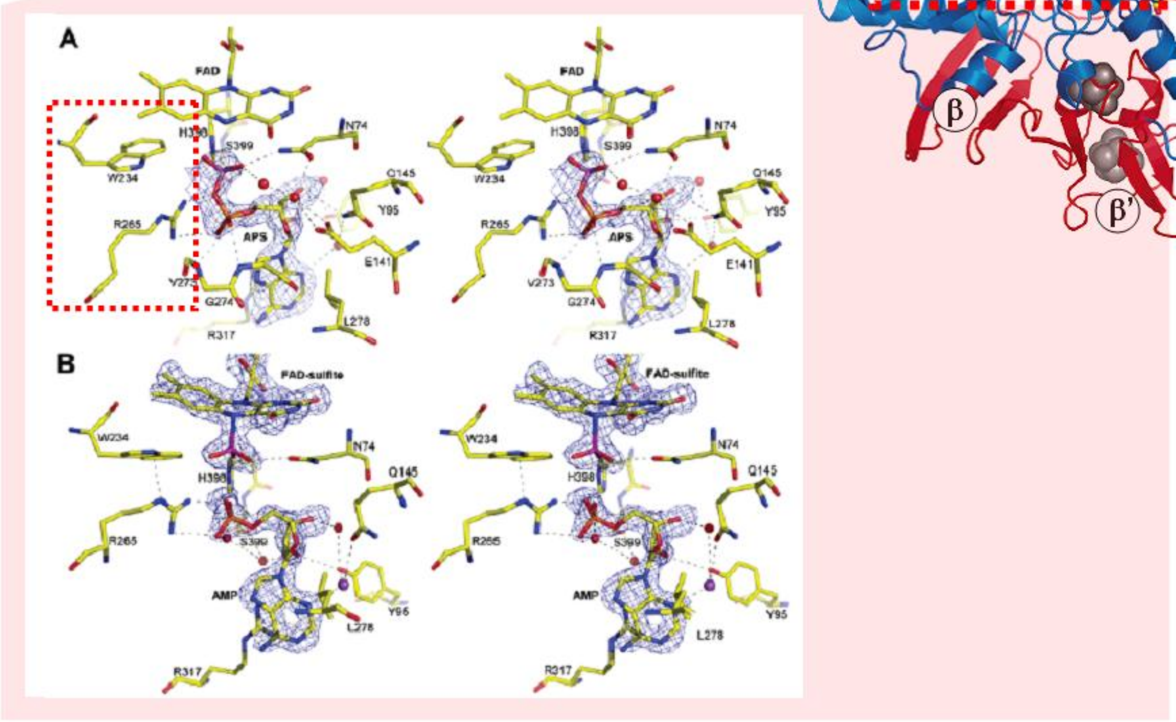

The iron-sulfur flavoenzyme adenosine-5'-phosphosulfate (APS) reductase (*Apr*) (EC: 1.8.99.2). *Apr* catalyzes a key reaction of the sulfur cycle by reversibly transforming APS to sulfite and AMP. The dissimilatory sulfate reduction operates under anaerobic conditions in *Bacteria* and *Archaea* (Schiffer et al., 2006). *Apr* consists of $\alpha_2\beta_2$ heterotetrameres (Fritz et al., 2002). The α-subunit (75 kDa) harbors the FAD cofactor. In the figure S2a, the *Apr* α-subunit is shown (blue) and the β-subunit (red). The evolutionary sequence region used in this work (see the alignment above) represents the N-terminal domain of the α–subunit (red rectangle), which is comprised of the [4Fe-4S] clusters and the FAD binding site (marked as ball-and-stick representations). The active site channel located between the central and capping domains of the α-subunit is filled with water molecules depicted as green spheres (Schiffer et al., 2006).

(A) *Apr* in the FADox-APS state. *Apr* binds in a curved conformation to the prebuilt channel. Invariant residues His-H$^{\alpha398}$, Asn-N$^{\alpha74}$ and Arg-R$^{\alpha265}$ play a key role in phosphosulfate binding and catalysis. The adenine part is firmly clamped between Leu-L$^{\alpha278}$ and Arg-R$^{\alpha317}$ and the latter swings into the channel upon substrate binding (Schiffer et al., 2006).

(B) *Apr* in the FAD-sulfite-AMP1 state. This state contains a FAD-sulfite adduct and AMP, the latter adopting a conformation similar to the AMP moiety of APS. This structure shows how the phosphate of AMP and the sulfite of the FAD-sulfite adduct (both negatively charged) are kept in van der Waals contact (Schiffer et al., 2006).

The binding affinity of APS for *Apr* is reflected by protein-substrate interactions. The sulfate O1 atom interacts with the ND2 atom of Asn-N$^{\alpha74}$, its O2 atom with the NE2 atom of His-H$^{\alpha389}$, and its O3 atom via a water molecule with Arg-R$^{\alpha265}$ and Trp-W$^{\alpha234}$ and via two water molecules with Glu-E$^{\alpha141}$, Asp-D$^{\alpha361}$ and Asn-N$^{\alpha74}$. Additionally, Arg-R$^{\alpha265}$ is hydrogen bonded to the O3A atom that links the sulfate and phosphate groups. The phosphate oxygen atoms are connected to Arg-R$^{\alpha265}$ via a salt bridge and to the peptide nitrogens of Val-V$^{\alpha273}$ and Gly-G$^{\alpha274}$, located at the positively charged N-terminus of helix R6. The hydroxyl group O2 of ribose is linked to the side chain hydroxyl group of Tyr-Y$^{\alpha95}$ and that of O3 to His-H$^{\alpha446}$ and Tyr-Y$^{\alpha95}$ via a water molecule. The fixation of the adenine ring of APS is mainly accomplished by sandwiching it between Leu-L$^{\alpha278}$ and Arg-R$^{\alpha317}$ whereby the coplanar arrangement of the guanidinium group is optimal for π-π interactions. Additionally, one polar contact is formed between the N3 atom of the adenine ring and the protein mediated by a solvent molecule (Schiffer et al., 2006).

Figure S1. b) Relative amino acid composition of the *AprA* catalytic region. The frequency of the 20 amino acids present in the alignment (above) was plotted across phylotypes as a range of percentages: between 0-5% (blue), 5-10% (red) and 10-15% (green).

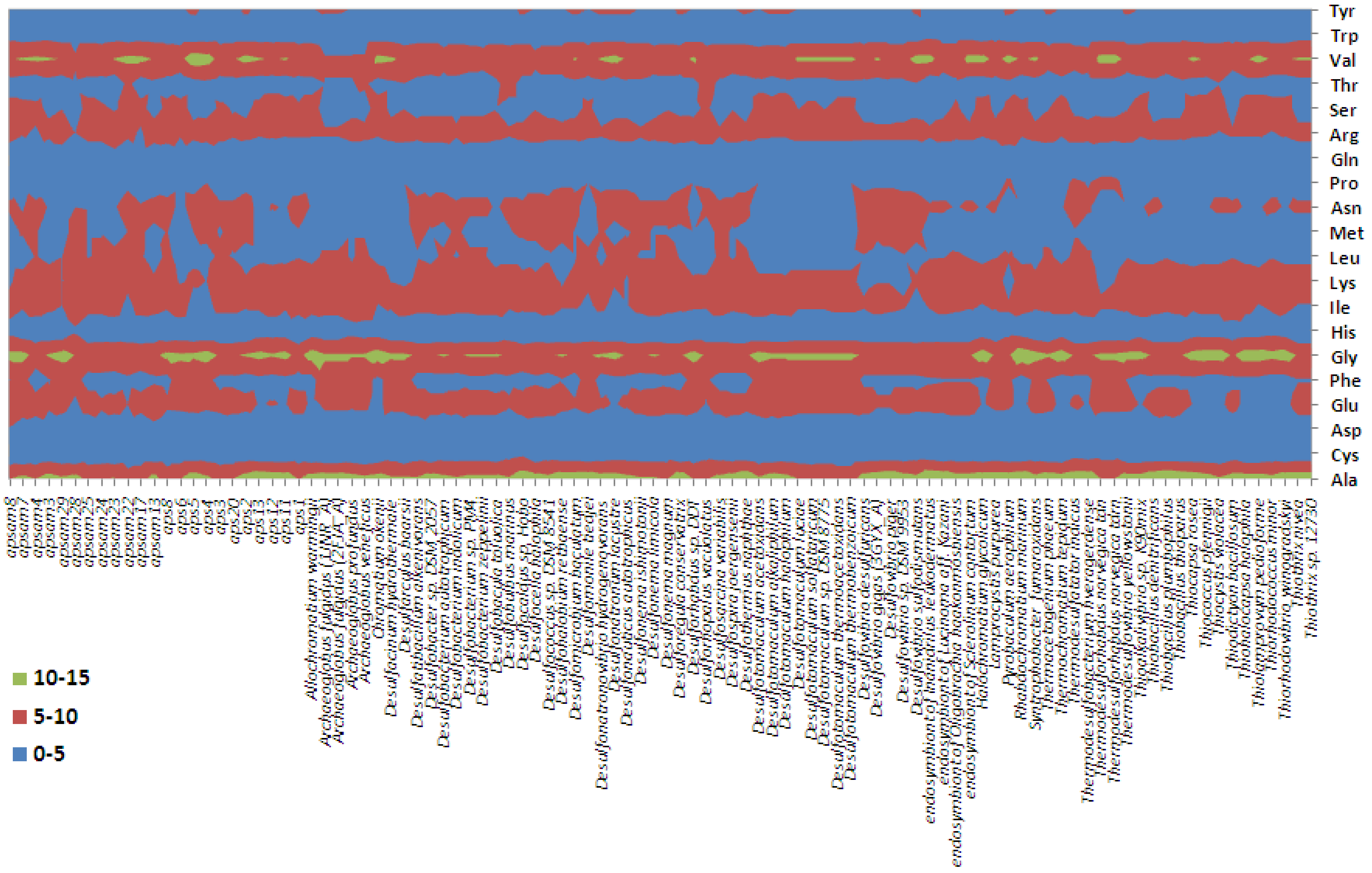

Figure S1. c) Nucleotide codon composition for the *aprA* marker in gene sequences used in this study.

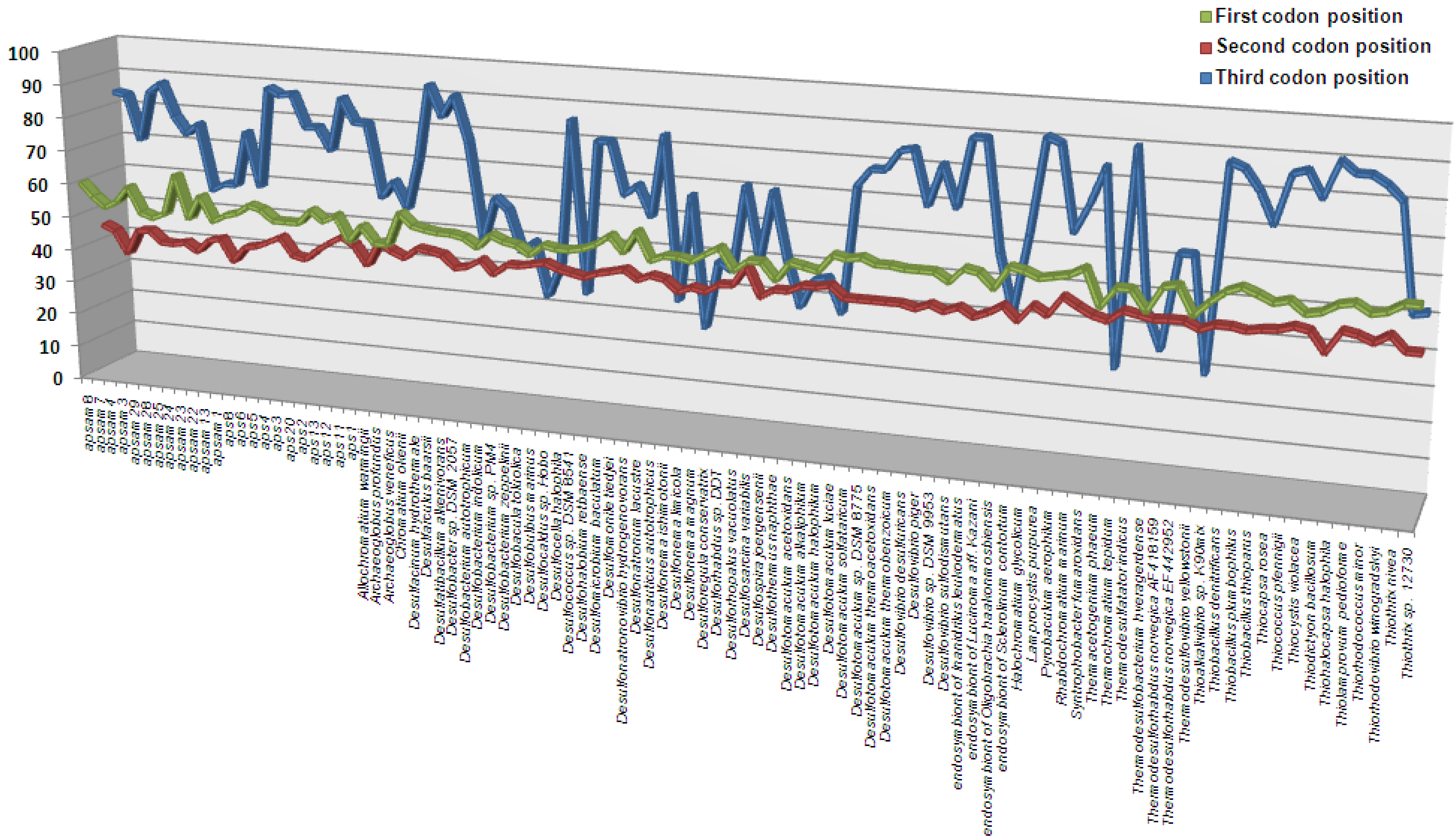

Figure S2. Alignment of 80 amino acid sequences with the corresponding fragment to the *McrA*, C-terminal domain. *McrA* C-terminal domain (from 273 to 551 amino acid positions) has been characterized from *Methanosarcina barkeri* (1E6Y) (Grabarse et al., 2000), *Methanothermobacter thermoautotrophicus* (1MRO) (Ermler et al., 1997) and *Methanopyrus kandlery* (1E6V) (Rospert et al., 1991). This alignment includes all of the functional active sites (marked with black background) of the Mcr_alpha_C domain: Phe-F$^{\alpha330}$, Tyr-Y$^{\alpha333}$, Phe-F$^{\alpha443}$, Tyr-Y$^{\alpha444}$, Gly-G$^{\alpha445}$, Asn-N$^{\alpha481}$ and Val-V$^{\alpha482}$ positions (Klein et al.) reported for *Mtb. thermoautotrophicus** (Ermler et al., 1997). **Metanothermobacter thermoautotrophicus*, new name for *Methanobacterium thermoautotrophicum* (Wasserfallen et al., 2000). Essential positions for amino acid modifications are marked with grey backgrounds as reported from PDB and UniProt databases. 132 from 139 positions were used for the phylogenetic reconstruction.

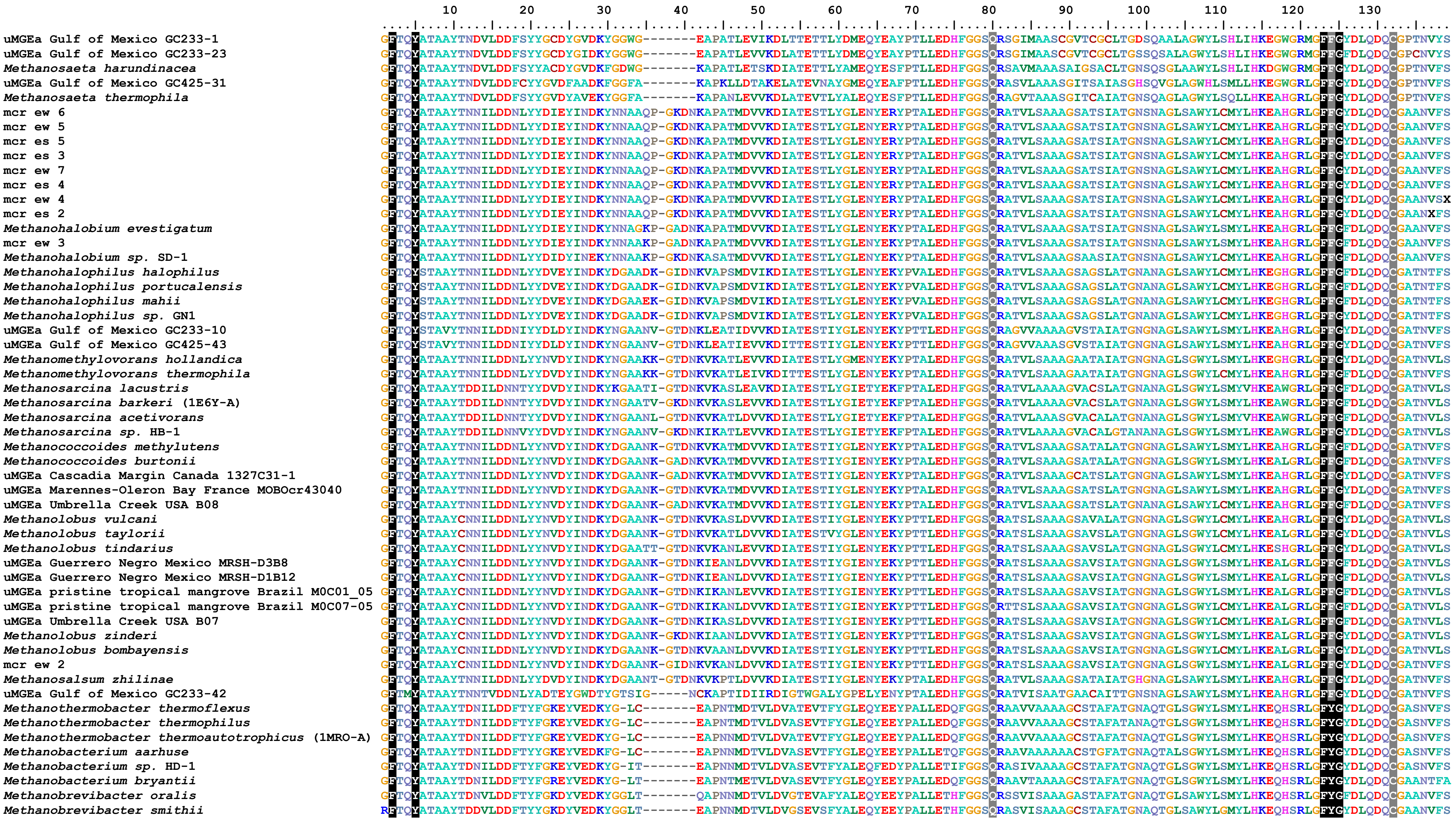

```
Methanothermus sociabilis                    GFTQYATASYTDNILDDFTYYGKEYVEDKYGGLT-------EAPHDFDTILDVATEVTLYSLEQYEEYPTLMEDHFGGSQRAAIAAAASGCSTAFATGNAQAGFSGWYLSQILHKEAHARLGFYGYDLQDQCGAANTYS
Methanothermus fervidus                      GFTQYATASYTDNILDDFTYYGKEYVEDKYGGLA-------EAPHDFDTILDVATEVTLYSLDQYEEYPTLMEDHFGGSQRAAIAAAASGCSTAFAAGNAQAGLSGWYLSQILHKECHARLGFYGYDLQDQCGAANTYS
Methanopyrus kandlery (1E6V-A)               GFTQYATAVYPDNILDDYVYYGLEYVEDKYG-IA-------EAEPSMDVVKDVATEVTLYGLEQYERYPAAMETHFGGSQRAAVCAAAAGCSTAFATGHAQAGLNGWYLSQILHKEGQGRLGFYGYALQDQCGAANSLS
Methanocaldococcus infernus                  GFTQYATASYTDDILDDFSYYGYDYINKKYGGCN-------SVKPTMDVVEDIAGEVTLYALEQYDTYPALLEDHFGGSQRRAVAAAASGISVALATGNSNAGLNGWYLSQIMHKEYHSRLGFYGYDLQDQCGAANSLS
Methanocaldococcus jannaschii                GFTQYATATYTDDILDDFSYYGYDYITKKYGGCN-------SVKPTMDVVEDIATEVTLYGLEQYDTFPALLEDHFGGSQRAGVTAAAAGITTALATGNSNAGVNGWYLSQILHKEYHSRLGFYGYDLQDQCGAANSLS
Methanococcus maripaludis                    GFTQYATASYTDDILDDFSYYALDYVEKKYGRMG--------TKATMDVVEDVAGEVTLYALEQYDDYPALLEDHFGGSQRAAVAAAASGIGVCMATGNSNAGVNGWYLSQILHKEYHSRLGFYGYDLQDQCGASNSLA
Methanococcus vannielii                      GFTQYATAAYTDDILDDFSYYALDYVEKKYGRMG--------TKATMDVVEDIASEVTLYSLEQYDEYPALLEDHFGGSQRAAVAAAASGIGVCMATGNSNAGVNGWYLSQILHKEYHSRLGFYGYDLQDQCGASNSLA
Methanococcus voltae                         GFTQYATASYTDDILDDFSYYGYEYVEKKYGRCG--------TKATMDVVEDIASEVTLYALEQYDEYPALLEDHFGGSQRAAVAAAAAGISVCMATGNSNAGVNGWYLSQILHKEYHSRLGFYGYDLQDQCGASNSLA
Methanothermococcus okinawensis              GFTQYATATYTDDILDDFSYYGYDYVEKKYGING--------TKPTMDVVEDIATEVTLYGLEQYDEFPALLEDHFGGSQRAGVVAAASGISVCMATGNSNAGVNGWYLSQILHKEYHSRLGFYGYDLQDQCGASNSLS
Methanococcus aeolicus                       GFTQYATAAYTDDILDDFCYYGLDYVEKKFGRCG--------TKATMDVVEDIATEVTLYSLERYDEYPTLLEDHFGGSQRAAVAAAAAGVSVCMATGNSNAGVNGWYLSQIMHKEYHSRLGFYGYDLQDQCGASNSLS
Methanococcus jannaschii                     GFTQYASATYTDDILDDFVYYGMDYVEKKYGLCG--------VKPSMEVVKDIATEVTLYGLEQYDEYPALLEDHFGGSQRAGVTAAAAGCSVAFATGNSNAGINGWYLSQILHKEYHSRLGFYGYDLQDQCGAANSLS
Methanocorpusculum labreanum                 GFTQYATAAYTDNILDDFVYXGMDYIHDKYKVDXKNPNPNDKVKATQEVVNDIATEVNLYGMEQYEQFPTMMEDHFGGSQRAAVLAAASGITTSIATGNSNAGLNGWYLSMLMHKDGWSRLGFFGYDLQDQCGSANSLS
Methanocorpusculum sp. MSP                   GFTQYATAAYTDNILDDFTYYGMDYIHDKYKIDTKSPNAKDKVKATQEVVNDIATEVNLYGMEQYEQFPTMMEDHFGGSQRAAVLAAASGISTSIATGNSNAGLNGWYLSMLLHKDGWSRLGFFGYDLQDQCGSANSLS
uMGEa Gulf of California Mexico D08          GFTQYATAAYTDNILDDFTYYGVDYLHDKYEIDWKNPNPKDKVKATQEVVNDIATEVNLYSMEQYEQFPTMMEDHFGGSQRAAVLGAACGLTTSIATGNSNAGLNGWYLSMLMHKDGWSRLGFFGYDLQDQCGSANSLS
Methanoculleus chikugoensis                  GFTQYATAAYTDNILDEFTYYGMDYIKDKYKVDWKNPSPKDKVKPTYDIVNDIATEVALNGMEQYEQYPTMMEDHFGGSQRAGVLAAACGLSTSIATGNSNAGLNAWYLCMLLHKDGWSRLGFFGYDLQDQCGSANSLS
uMGEa Gulf of California Mexico H07          GFTQYATAAYTDNILDEFTYYGMDYIKDKYKVDWKNPSPDDKVKPTQEIVNDIATEVALNGMEQYEQYPTMMEDHFGGSQRAGVLAAACGLSTSIATGNSNAGLNGWYLCMLLHKDGWSRLGFFGYDLQDQCGSANSLS
Methanoculleus sp. M11                       GFTQYATAAYTDNILDEFTYYGMDYIKDKYKVDWKNPSADDKVKPTYDIVNDISTEVALNGMEQYEQYPTMMEDHFGGSQRAGVLAAASGLSASIATGNSNAGLNAWYLCMLLHKDGWSRLGFFGYDLQDQCGSANSLS
uMGEa Gulf of California Mexico C10          GFTQYATAAYTDNILDEFTYYGMDYIKDKYKVDWKNPSPDDRVKPTQEVVNDIATEVALNGMEQYEQFPTMMEDHFGGSQRAGVLAAACGLSSSIATGNSNAGLNGWYLCMLLHKDGWSRLGFFGYDLQDQCGSANTLS
Methanoculleus thermophilus                  GFTQYATAAYTDNILDEFTYYGMDYIKDKYKVDWKNPSPSDKVKPTQDIVNDIATEVTLNAMEQYEQFPTMMEDHFGGSQRAGVIAAASGLSTSIATGNSNAGLNGWYLSMLLHKDGWSRLGFFGYDLQDQCGSANSLS
Methanoculleus palmolei                      GFTQYATAAYTDNILDEFTYYGMDYIKDKYKVDWKNPSPKDKVKPTQEIVNDMATEVTLNAMEQYEQFPTMMEDHFGGSQRAGVIAAASGLTTSIATGNSNAGLNGWYLSMLLHKDGWSRLGFFGYDLQDQCGSANSLS
Methanoculleus bourgensis                    GFTQYATAAYTDNILDEFTYYGMDYIKDKYKVDWKNPSAKDKIKPTQDVVNDMATEVTLNAMEQYEQFPTMMEDHFGGSQRAGVIAAASGLTTAIATGNSNAGLNGWYLSMLLHKDGWSRLGFFGYDLQDQCGSANSLS
Methanoplanus petrolearius                   GFTQYATAAYTDNILDEFTYYGMDYIGDKYNVDWKNPSPSDKIKPTQDIVNDIATEVCLNAMEQYEQFPTMMEDHFGGSQRAGVMAAACGLSTSIATGNSNAGLNAWYLCMLMHKDGWSRLGFFGYDLQDQCGSANSLS
mcr ew 1                                     GFTQYATAAYTDNILDEFTYYGMDYIKQKYKVDWQNPNEKDKVKPTQDIVNDIATEVCLNGMEQYEQFPTMMEDHFGGSQRAGVLAAACGLSTSIATGNSNAGLNAWYLCMLMHKEGWSRLGFFGYDLQDQCGSANSLA
uMGEa Umbrella Creek USA D04                 GFTQYATAAYTDNILDEYTYYGMDYIKQKYKVDWQNPNEKDKVKPTQEIVNDMCTEVCLNAMEQYEQFPTLMEDHFGGSQRAGVMAAACGLTASIATGYSNAGLNSWYLCMLMHKEGWSRLGFFGYDLQDQCGSANSLA
uMGEa Florida Everglades USA MCR-F1SP-2       DFTQYATAAYTDNILDEYTYYGMDYIKDKYKVDWKNPNEKDKVKPTQDIVNDMATEVALNGMEQYEQFPTLMEDHFGGSQRAGVLAAACGLTTSIATGNSNAGLNAWYLCMLLHKEGWSRLGFFGYDLQDQCGSANSLA
uMGEa Gulf of Mexico GC233-5                  GFTQYATAAYTDNILDEFTYYGMDYVKDKYGYDYTAPATDASVTPTQDIVNDITTEVNLNAMEQYEQFPTLMEDHFGGSQRAGVIAAACGLSTSIPTGNSNAGLSAWYLSMLMHKEGWSRLGFFGYDLQDQCGSANSLS
Clustal Consensus                             ** *::* * :: :*:  *   ::  ..:                  :    ::          : :: :*. :*  ******    .*: .       :. .: .. .*:*   :**:  .*:**:*: ******. *
```

Figure S2. a) Description of the catalytic sites for *McrA*.

Methyl–coenzyme M reductase (*Mcr*) (EC: 2.8.4.1) is a key enzyme in formation in methanogenic *Archaea*. It catalyzes the reduction of methyl–coenzyme M (methyl-CoM) with coenzyme B (Anderson et al.) to methane and the heterodisulfide of CoM and CoB (Ermler et al., 1997). This reaction proceeds under anaerobic conditions and requires a nickel-porphinoid prosthetic group, coenzyme F_{430}, which is in the EPR-detectable Ni (I) oxidation state in the active enzyme. *Mcr* is a 300 kDa hexamer protein arranged as $\alpha 2\beta 2\gamma 2$ subunits with two identical nickel porphinoid active sites, which form two long active site channels with F_{430} embedded at the bottom (Grabarse et al., 2000). The evolutionary sequence region used in this work (see the alignment above) represents the C-terminal domain of the α-subunit (red rectangle), which is comprised of an all-alpha multi-helical bundle (blue and turquoise helices).

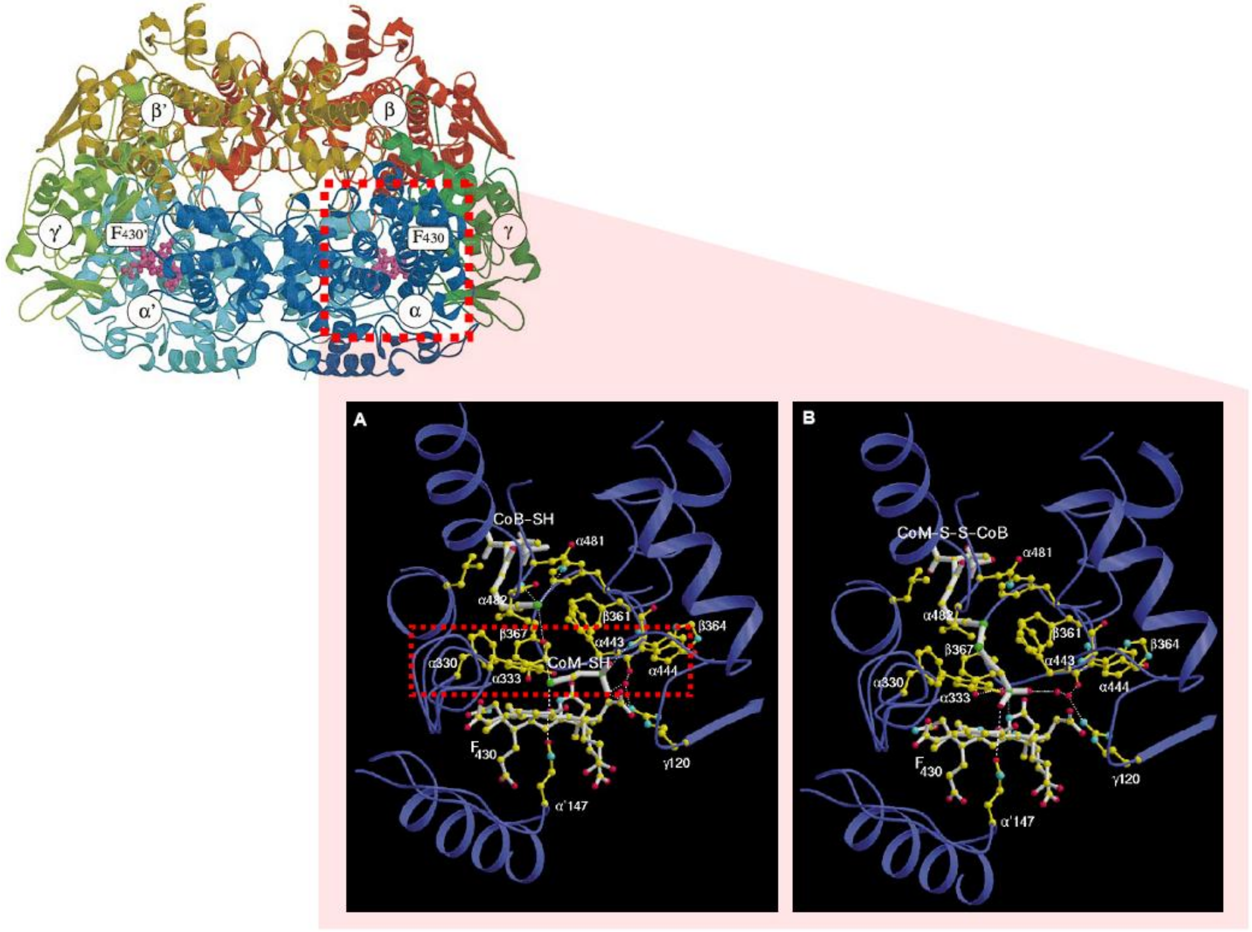

(A) The active site region of the $Mcr_{ox1\text{-}silent}$ structure. The binding positions of the coenzymes suggest the active site between the nickel of coenzyme F_{430} and the sulfur atom of CoB. The active site is coated mostly by non-polar and aromatic residues. Five mutually contacting Phe and Tyr side chains are arranged as ring forming a tunnel (Ermler et al., 1997).

(B) The active site region of the *Mcr* silent structure. *C*ompared with the $Mcr_{ox1\text{-}silent}$ structure, CoM has moved through the tunnel to form with CoB a heterodisulfide, the oxidation product of the reaction. The sulfonate moiety of CoM lost its interactions to the protein matrix and is coordinated to the Ni atom (Ermler et al., 1997).

The relative arrangement of the three coenzymes (CoM, CoB and CoF_{430}) suggests that the catalytic reaction takes place at the front side of CoF_{430} in the channel between the nickel and the thiol groups of CoB. Each active site is lined up by an annular arrangement of Phe-$F^{\alpha330}$, Tyr-$Y^{\alpha333}$, Phe-$F^{\alpha443}$, Phe-$F^{\beta361}$ and Tyr-$Y^{\beta367}$ flanked by further hydrophobic and aromatic residues. As can be seen in the alignment above, the Phe-$F^{\alpha330}$, Tyr-$Y^{\alpha333}$, Phe-$F^{\alpha443}$ amino acids are completely conserved in all *Mcr* sequences included in this work. Other studies on a catalytically inactive enzyme aerobically co-crystallized with CoM displayed a fully occupied CoM-binding site with no alternate conformations. The binding of CoM appears to induce specific conformational changes that suggest a molecular mechanism by which the enzyme ensures that methyl-coenzyme M enters the substrate channel prior to CoB, as required by the active-site geometry (Ermler et al., 1997).

Figure S2. b) Relative amino acid composition of the *McrA* catalytic region. The frequency of the 20 amino acids present in the alignment (above) was plotted across phylotypes as a range of percentages: between 0-5% (blue), 5-10% (red), 10-15% (green) and 15-20% (violet).

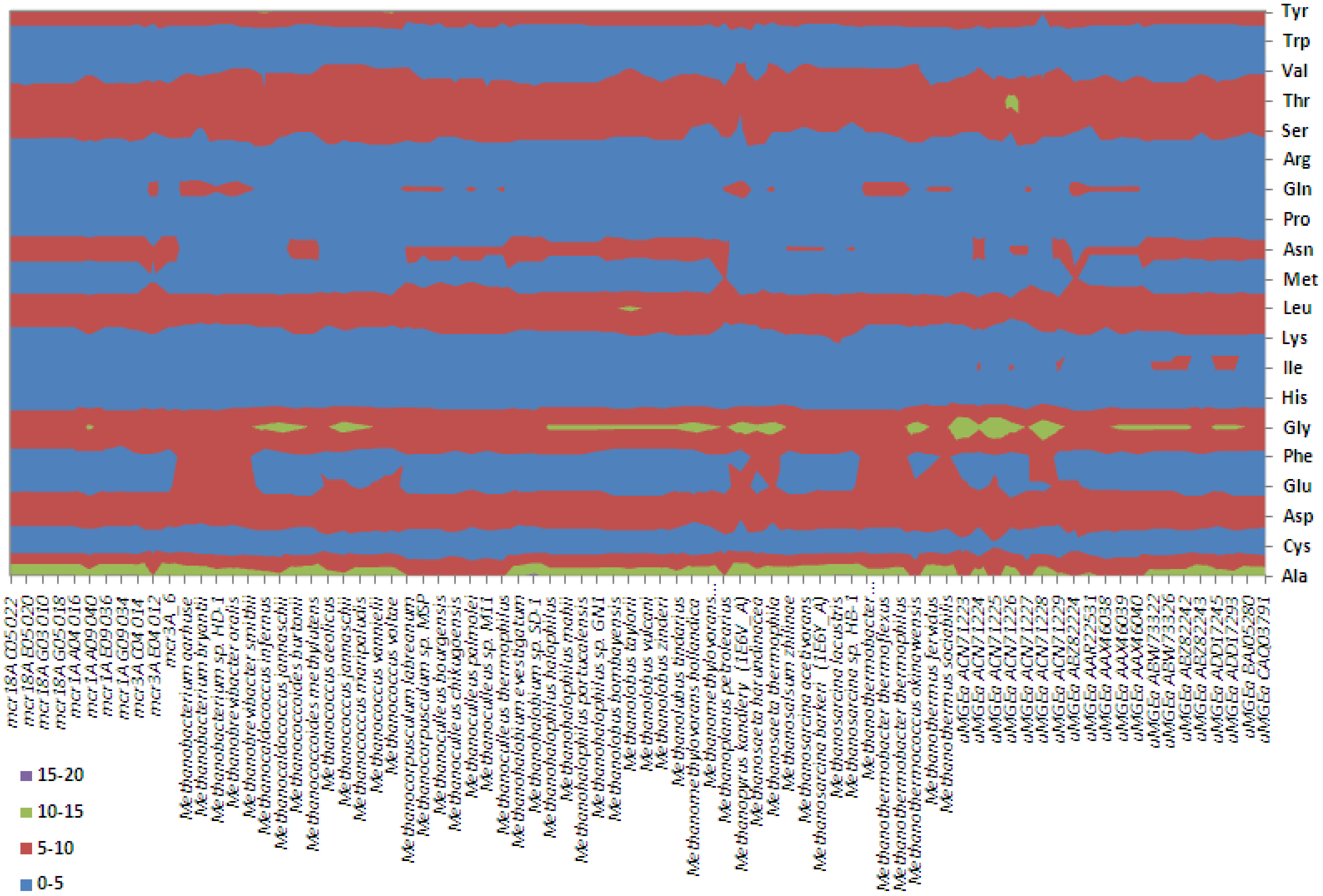

Figure S2. c) Nucleotide codon composition for the *mcrA* marker in gene sequences used in this study.

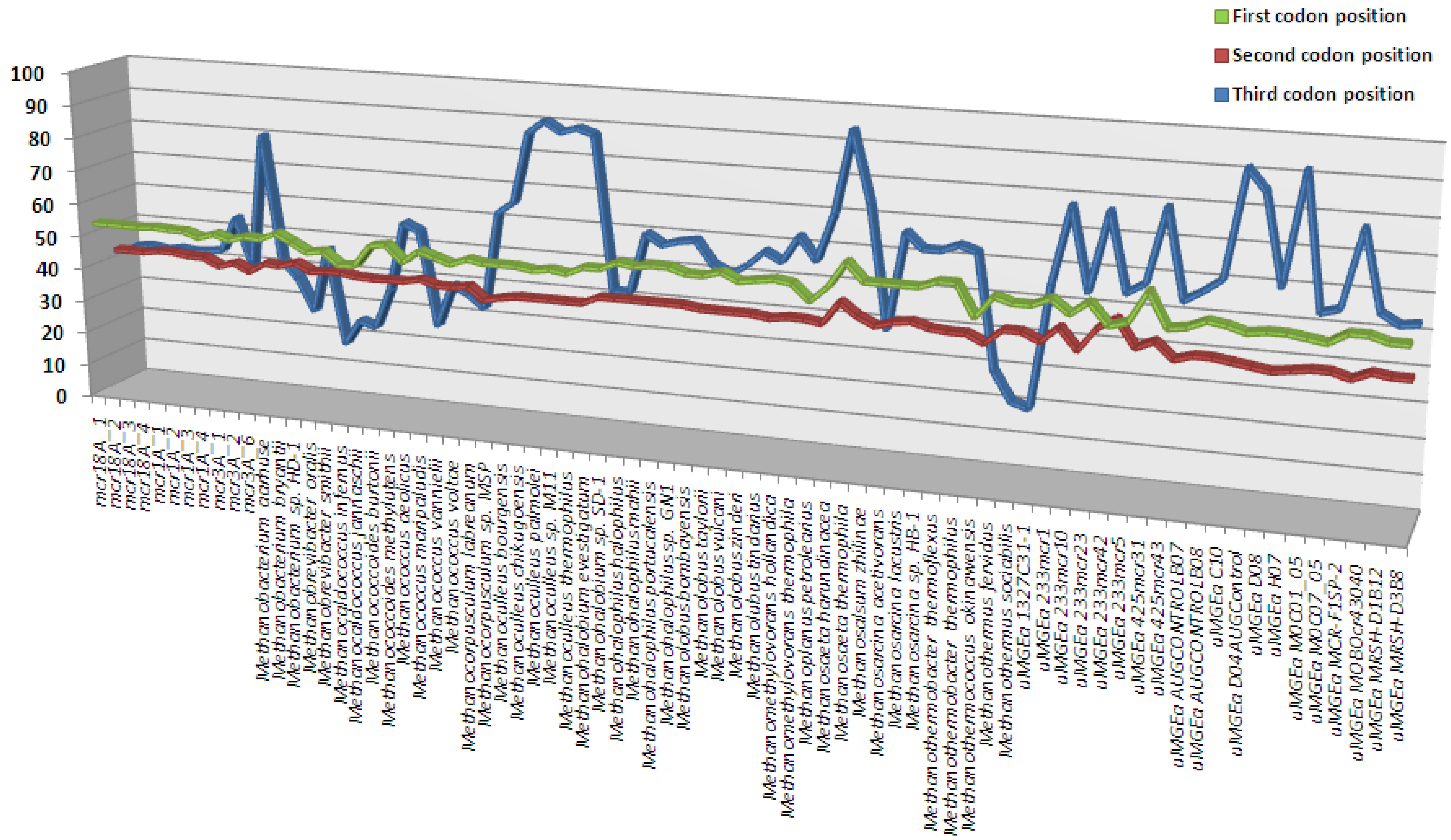

Figure S3. Correspondence analysis of Relative Synonymous Codon Usage (RSCU) for McrA sequences from halophiles, non-halophiles and Tirez phylotypes. The distribution of all codons (including the start and stop codons *) for every amino acid across the three datasets is shown on the X axis. The frequency of each codon (%) is represented with bars on the left Y axis. RSCU values for each codon across the three datasets are represented with differentiated dots on the right Y axis. In the absence of any codon usage bias, the RSCU value would be 1.00. A codon that is used less frequently than expected will have a value of less than 1.00 and *vice versa* for a codon that is used more frequently than expected.

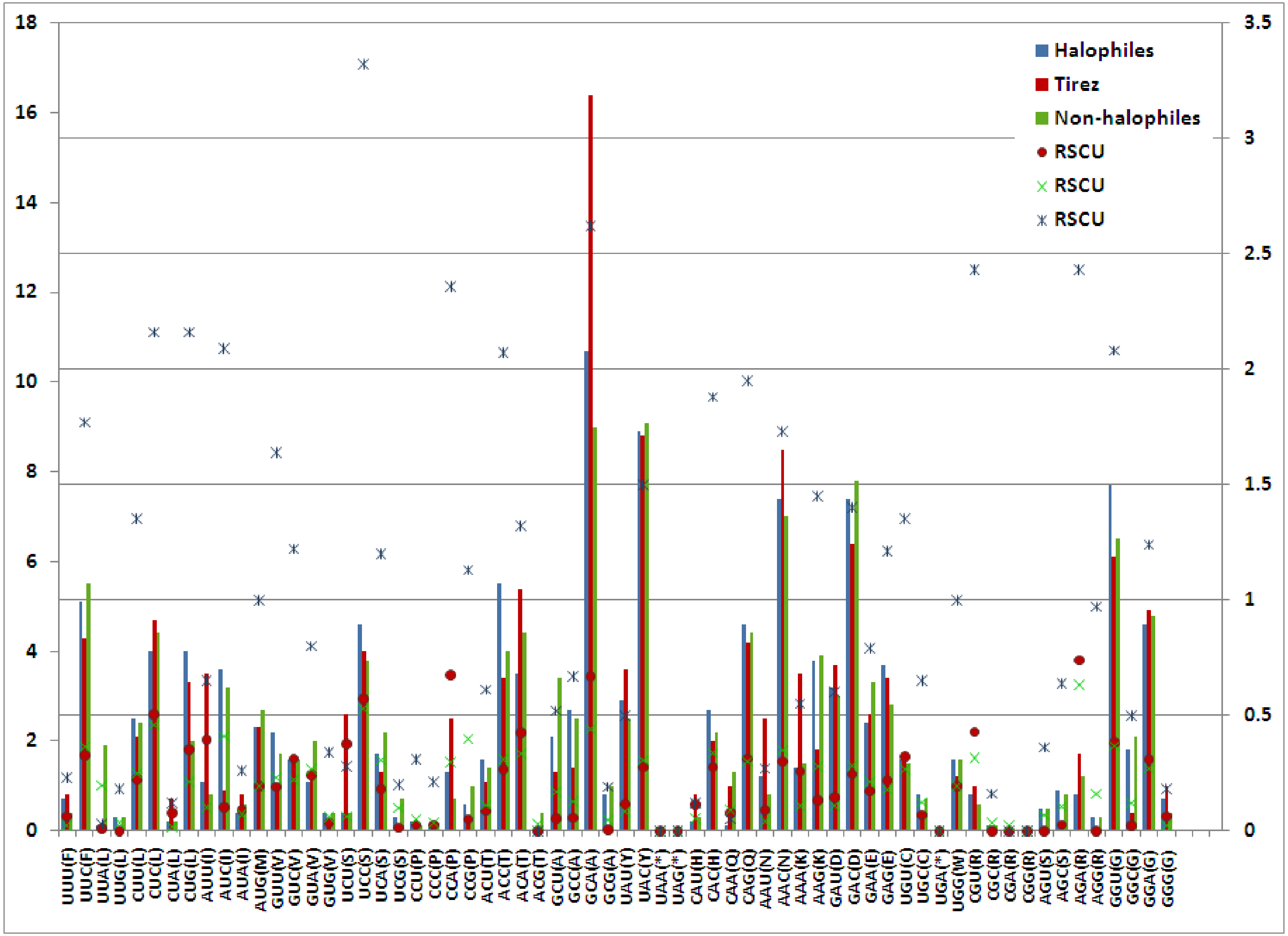

Figure S4. Diagram of the target sites for primer pairs within *mcr*. Modified from (Hales et al., 1996)

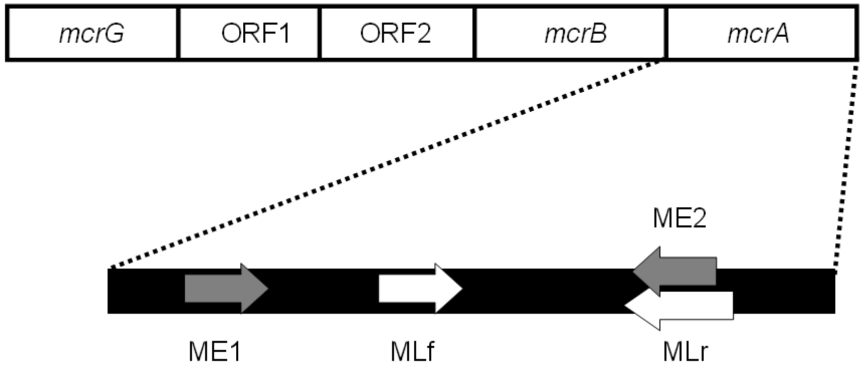

References for Supplementary Material

ANDERSON, J. D., SCHUBERT, G., JACOBSON, R. A., LAU, E. L., MOORE, W. B. & SJOGREN, W. L. 1998. Europa's Differentiated Internal Structure: Inferences from Four Galileo Encounters. *Science,* 281**,** 2019-2022.

CHIANG, Y. L., HSIEH, Y. C., FANG, J. Y., LIU, E. H., HUANG, Y. C., CHUANKHAYAN, P., JEYAKANTHAN, J., LIU, M. Y., CHAN, S. I. & CHEN, C. J. 2009. Crystal structure of Adenylylsulfate reductase from *Desulfovibrio gigas* suggests a potential self-regulation mechanism involving the C terminus of the beta-subunit. *J Bacteriol,* 24**,** 7597-7608.

ERMLER, U., GRABARSE, W., SHIMA, S., GOUBEAUD, M. & THAUER, R. K. 1997. Cristal structure of Methyl Coenzyme M Reductase: the key enzyme of biological methane formation. *Science,* 278**,** 1457-1462.

FRITZ, G., ROTH, A., SCHIFFER, A., BÜCHERT, T., BOURENKOV, G., BARTUNIK, H. D., HUBER, H., STETTER, K. O., KRONECK, P. M. H. & ERMLER, U. 2002. Structure of adenylylsulfate reductase from the hyperthermophilic *Archaeoglobus fulgidus* at 1.6-Å resolution. *Proc Natl Acad Sci USA,* 99**,** 1836-1841.

GRABARSE, W., MAHLERT, F., SHIMA, S., THAUER, R. K. & ERMLER, U. 2000. Comparison of three methyl-coenzyme M reductases from phylogenetically distant organisms: unusual amino acid modification, conservation and adaptation. *J Mol Biol,* 303**,** 329-344.

HALES, B. A., EDWARDS, C., RITCHIE, D. A., HALL, G., PICKUP, R. W. & SAUNDERS, J. R. 1996. Isolation and Identification of Methanogen-Specific DNA from Blanket Bog Peat by PCR Amplification and Sequence Analysis. *Appl Environ Microbiol,* 62**,** 668-675.

KLEIN, M., FRIEDRICH, M., ROGER, A. J., HUGENHOLTZ, P., FISHBAIN, S., ABICHT, H., BLACKALL, L. L., STAHL, D. A. & WAGNER, M. 2001. Multiple Lateral Transfers of Dissimilatory Sulfite Reductase Genes between Major Lineages of Sulfate-Reducing Prokaryotes. *J Bacteriol,* 183**,** 6028-6035.

OREN, A. & MANA, L. 2002. Amino acid composition of bulk protein and salt relationships of selected enzymes of *Salinibacter rubber*, an extremely halophilic bacterium. *Extremophiles* 6**,** 217-223.

RHODES, M. E., FITZ-GIBBON, S. T., OREN, A. & HOUSE, C. H. 2010. Amino acid signatures of salinity on an environmental scale with a focus on the Dead Sea. *Environ Microbiol,* 12**,** 2613-23.

ROSPERT, S., BREITUNG, J., MA, K., SCHWÖRER, B., SIRNGIBL, C., THAUER, R. K., LINDER, D., HUBER, R. & STETTER, K. O. 1991. Methyl-coenzyme M reductase and other enzymes involved in methanogenesis from CO2 and H2 in the extreme thermophile *Methanopyrus kandlery*. *Arch Microbiol,* 156**,** 49-55.

SCHIFFER, A., FRITZ, G., KRONECK, P. M. H. & ERMLER, U. 2006. Reaction Mechanism of the Iron−Sulfur Flavoenzyme Adenosine-5'-Phosphosulfate Reductase Based on the Structural Characterization of Different Enzymatic States. *Biochemistry,* 45**,** 2960-2967.

TAJIMA, F. 1989. Statistical methods to test for nucleotide mutation hypothesis by DNA polymorphism. *Genetics,* 123**,** 585-595.

TAMURA, K., DUDLEY, J., NEI, M. & KUMAR, S. 2007. MEGA 4: Molecular Evolutionary Genetics Analysis (MEGA) software version 4.0. *Mol Biol Evol,* 24**,** 1596-1599.

WASSERFALLEN, A., NOLLING, J., PFISTER, P., REEVE, J. & CONWAY DE MACARIO, E. 2000. Phylogenetic analysis of 18 thermophilic Methanobacterium isolates supports the proposals to create a new genus, Methanothermobacter gen. nov., and to reclassify several isolates in three species, Methanothermobacter thermautotrophicus comb. nov., Methanothermobacter wolfeii comb. nov., and Methanothermobacter marburgensis sp. nov. *Int J Syst Evol Microbiol,* 50**,** 43-53.
